# European Network for Optimization of Veterinary Antimicrobial Therapy (ENOVAT) 2025 guidelines for surgical antimicrobial prophylaxis in dogs and cats

**DOI:** 10.1111/jsap.70072

**Published:** 2025-12-23

**Authors:** F. Allerton, T. M. Sørensen, K. Scahill, J. E. Ruperez, F. Swinbourne, D. R. Verwilghen, M. C. Nolff, F. Foroutan, S. J. Baines, A. Vilen, L. Pelligand, E. M. Broens, P. L. Toutain, M. L. Brennan, T. Mooney, S. Clarke, J. E. Miles, J. L. Granick, Y. Winsborg, L. R. Jessen, J. S. Weese, the European Network for Optimization of Veterinary Antimicrobial Therapy (ENOVAT) guidelines and the ESCMID Study Group for Veterinary Microbiology (ESGVM)

**Affiliations:** ^1^ Willows Veterinary Centre & Referral Service (Part of Linnaeus Veterinary Limited) Solihull UK; ^2^ Department of Veterinary Clinical Sciences University of Copenhagen Frederiksberg C Denmark; ^3^ Department of Pathobiology, Ontario Veterinary College University of Guelph Guelph Ontario Canada; ^4^ College of Medicine and Veterinary Medicine University of Edinburgh Edinburgh UK; ^5^ Evidensia Södra Djursjukhuset Kungens Kurva Kungens Kurva Sweden; ^6^ Department of Small Animal Surgery, Section of Small Animal Clinical Studies University College Dublin Dublin Ireland; ^7^ Lumbry Park Veterinary Specialists Alton UK; ^8^ Sydney School of Veterinary Science University of Sydney Camperdown New South Wales Australia; ^9^ Clinic for Small Animal Surgery, Vetsuisse Faculty University Zürich Zürich Switzerland; ^10^ Faculty of Health Sciences McMasters University Hamilton Ontario Canada; ^11^ AniCura Landskrona Smådjursklinik Landskrona Sweden; ^12^ Department of Comparative Biomedical Sciences Royal Veterinary College London UK; ^13^ Department of Clinical Sciences and Services Royal Veterinary College London UK; ^14^ ESCMID Study Group for Veterinary Microbiology (ESGVM) Basel Switzerland; ^15^ Faculty of Veterinary Medicine Utrecht University Utrecht The Netherlands; ^16^ INTHERES, INRAE, ENVT Université de Toulouse Toulouse France; ^17^ Centre for Evidence‐Based Veterinary Medicine The University of Nottingham Loughborough UK; ^18^ Animal Trust CIC Dewsbury UK; ^19^ Ferguson Veterinary Clinic Glenrothes UK; ^20^ Veterinary Clinical Sciences Department University of Minnesota College of Veterinary Medicine St. Paul Minnesota USA

## Abstract

Surgical antimicrobial prophylaxis involves the administration of antimicrobials to reduce the risk of a surgical site infection and represents a significant proportion of all antimicrobial use in cats and dogs. This evidence‐based, European Network for Optimization of Veterinary Antimicrobial Therapy guideline provides recommendations for both peri‐ and post‐operative surgical antimicrobial prophylaxis for a wide range of soft tissue and orthopaedic procedures performed in dogs and cats. A multidisciplinary panel developed the recommendations while adhering to the Grading of Recommendations Assessment, Development and Evaluation framework. The opinions of veterinary practitioners were incorporated to ensure applicability. Ten strong recommendations against, three conditional recommendations against and five conditional recommendations for the use of surgical antimicrobial prophylaxis were drafted by the panel. Strong recommendations against surgical antimicrobial prophylaxis were often informed by low‐ to very low‐certainty evidence that treatment has no beneficial effect. However, the anticipated harmful effects of antimicrobial use are well established and offer an important counterbalance to unsubstantiated use. Conditional recommendations were made when there was a probable balance of effects in one direction, although appreciable uncertainty was present. The European Network for Optimization of Veterinary Antimicrobial Therapy guidelines initiative encourages national or regional guideline makers to use the evidence presented in this document and the supporting systematic review to draft national or local guidance documents that support rational surgical antimicrobial prophylaxis.

Executive summary (recommendations and remarks without full rationale)

**Table:** 

**Recommendation 1 – Peri‐operative antimicrobial use in neutering** In dogs and cats undergoing neutering, we recommend against the use of peri‐operative surgical antimicrobial prophylaxis *Strong recommendation, low‐certainty evidence. Level of agreement 100% (17/17)*
**Recommendation 2 – Post‐operative antimicrobial use in neutering** In dogs and cats undergoing neutering, we recommend against the use of post‐operative surgical antimicrobial prophylaxis *Strong recommendation, low‐certainty evidence. Level of agreement 100% (17/17)*
**Recommendation 3 – Peri‐operative antimicrobial use in other clean procedures** In dogs and cats undergoing clean soft tissue surgical procedures other than neutering (e.g. splenectomy, dermal mass removal, exploratory laparotomy, gastropexy), we recommend against the use of peri‐operative surgical antimicrobial prophylaxis *Strong recommendation, very low‐certainty evidence. Level of agreement 100% (17/17)*
**Recommendation 4 – Post‐operative antimicrobial use in other clean procedures** In dogs and cats undergoing clean soft tissue surgical procedures other than neutering (e.g. splenectomy, dermal mass removal, exploratory laparotomy, gastropexy), we recommend against the use of post‐operative surgical antimicrobial prophylaxis *Strong recommendation, very low‐certainty evidence. Level of agreement 100% (17/17)*
**Recommendation 5 – Peri‐operative antimicrobial use in clean‐contaminated urologic procedures** In dogs and cats undergoing clean‐contaminated urological surgical procedures, we suggest not to use peri‐operative surgical antimicrobial prophylaxis *Conditional recommendation, very low‐certainty evidence. Level of agreement 100% (17/17)*
**Recommendation 6 – Post‐operative antimicrobial use in clean‐contaminated urologic procedures** In dogs and cats undergoing clean‐contaminated urological surgical procedures, we recommend against the use of post‐operative surgical antimicrobial prophylaxis *Strong recommendation, very low‐certainty evidence. Level of agreement 100% (17/17)*
**Recommendation 7 – Peri‐operative antimicrobial use in clean‐contaminated gastrointestinal procedures** In dogs and cats undergoing clean‐contaminated gastrointestinal surgical procedures, we suggest administration of peri‐operative surgical antimicrobial prophylaxis *Conditional recommendation, very low‐certainty evidence. Level of agreement 94% (16/17)*
**Recommendation 8 – Post‐operative antimicrobial use in clean‐contaminated gastrointestinal procedures** In dogs and cats undergoing clean‐contaminated gastrointestinal surgical procedures, we recommend against the use of post‐operative surgical antimicrobial prophylaxis *Strong recommendation, very low‐certainty evidence. Level of agreement 100% (17/17)*
**Recommendation 9 – Peri‐operative antimicrobial use in other clean‐contaminated procedures** In dogs and cats undergoing clean‐contaminated surgical procedures, not involving the urological or gastrointestinal tracts (e.g. corrective interventions for brachycephalic obstructive airway syndrome), we recommend against the use of peri‐operative surgical antimicrobial prophylaxis *Strong recommendation, very low‐certainty evidence. Level of agreement 100% (17/17)*
**Recommendation 10 – Post‐operative antimicrobial use in other clean‐contaminated procedures** In dogs and cats undergoing clean‐contaminated surgical procedures not involving the urological or gastrointestinal tracts (e.g. corrective interventions for brachycephalic obstructive airway syndrome), we recommend against the use of post‐operative surgical antimicrobial prophylaxis *Strong recommendation, very low‐certainty evidence. Level of agreement 100% (17/17)*
**Recommendation 11 – Peri‐operative antimicrobial use in contaminated procedures** In dogs and cats undergoing contaminated soft tissue surgical procedures, we suggest administration of peri‐operative surgical antimicrobial prophylaxis *Conditional recommendation, very low‐certainty evidence. Level of agreement 100% (17/17)*
**Recommendation 12 – Post‐operative antimicrobial use in contaminated procedures** In dogs and cats undergoing contaminated soft tissue surgical procedures, we suggest that post‐operative surgical antimicrobial prophylaxis should be used for 3 to 5 days with daily review and adaptation/de‐escalation in line with culture and susceptibility test results *Conditional recommendation, very low‐certainty evidence. Level of agreement 88% (15/17)*
**Recommendation 13 – Peri‐operative antimicrobial use in non‐implant orthopaedic procedures** In dogs and cats undergoing clean orthopaedic surgical procedures not involving the placement of an implant, we suggest not to use peri‐operative surgical antimicrobial prophylaxis *Conditional recommendation, very low‐certainty evidence. Level of agreement 100% (17/17)*
**Recommendation 14 – Post‐operative antimicrobial use in non‐implant orthopaedic procedures** In dogs and cats undergoing clean orthopaedic surgical procedures not involving the placement of an implant, we recommend against the use of post‐operative surgical antimicrobial prophylaxis *Strong recommendation, low‐certainty evidence. Level of agreement 100% (17/17)*
**Recommendation 15 – Peri‐operative antimicrobial use in implant orthopaedic procedures** In dogs and cats undergoing clean orthopaedic surgical procedures involving the placement of an implant, we suggest administration of peri‐operative surgical antimicrobial prophylaxis *Conditional recommendation, very low‐certainty evidence. Level of agreement 100% (17/17)*
**Recommendation 16 – Post‐operative antimicrobial use in implant orthopaedic procedures** In dogs and cats undergoing clean orthopaedic surgical procedures involving the placement of an implant, we recommend against the use of post‐operative surgical antimicrobial prophylaxis *Strong recommendation, moderate‐certainty evidence. Level of agreement 100% (17/17)*
**Recommendation 17 – Peri‐operative antimicrobial use in TPLO procedures** In dogs undergoing a Tibial Plateau Levelling Osteotomy (TPLO) procedure, we suggest administration of peri‐operative surgical antimicrobial prophylaxis *Conditional recommendation, very low‐certainty evidence. Level of agreement 94% (16/17)*
**Recommendation 18 – Post‐operative antimicrobial use in TPLO procedures** In dogs undergoing a Tibial Plateau Levelling Osteotomy (TPLO) procedure, we suggest not to use post‐operative surgical antimicrobial prophylaxis *Conditional recommendation, very low‐certainty evidence. Level of agreement 100% (17/17)*

## INTRODUCTION

Surgical site infections (SSIs) compromise pet health, potentially necessitate additional intervention including corrective surgical procedures and/or treatment (including antimicrobial use), lead to increased costs for caregivers and can contribute to increased mortality. Multiple clinical practice guidelines have been produced to guide surgical antimicrobial prophylaxis (SAP) and prevent SSIs in human medicine (Allegranzi et al., 2016; Ban et al., 2017; Berríos‐Torres et al., 2017; Bratzler et al., 2013; Sartelli et al., 2024). Such guidelines rely on a critical evaluation of the available literature through systematic reviews and meta‐analyses to assess the impact of peri‐operative measures, including SAP, on SSI risk (Hassan et al., 2021; Liu et al., 2018). However, while national and international consensus‐based SAP recommendations are included in multiple antimicrobial use guidelines in veterinary medicine (Allerton et al., 2021), evidence‐based guidance that balances benefits and harms based on systematic review and meta‐analysis regarding SAP in dogs and cats is not available, even though SAP accounts for around 15% of all antibiotic use in dogs and cats (Hsieh et al., 2022).

A recent scoping review (Sørensen et al., 2024) identified 34 studies (8 randomised controlled trials (RCTs), 7 prospective and 16 retrospective cohort studies and 3 retrospective case series) that contained data relating to SSI rates with or without SAP. A certainty assessment of the RCTs and meta‐analysis of both the RCTs and prospective observational studies (Sørensen et al., under review) has been performed to inform these European Network for Optimization of Antimicrobial Therapy (ENOVAT) SAP Guidelines.

## SCOPE AND PURPOSE

This document offers guidance to veterinary practitioners performing soft tissue and/or orthopaedic surgical procedures, on the use of SAP in dogs and cats, based on the available evidence and transparent reasoning. These guidelines provide separate recommendations for both peri‐ and post‐operative SAP (defined below) and are intended to help practitioners determine when SAP could significantly improve SSI outcomes and where it should be avoided because the anticipated harms outweigh the benefits. The ENOVAT guidelines initiative encourages national or regional guideline makers to use the evidence presented here to draft national or local recommendations. Translation and dissemination of ENOVAT guideline documents is also supported. This guideline is produced in collaboration with the European Society of Clinical Microbiology and Infectious Disease (ESCMID) Study Group for Veterinary Microbiology (ESGVM).

## METHODS

This guideline was produced following the ENOVAT operating procedure (https://enovat.eu/). The Grading of Recommendations, Assessment, Development and Evaluation (GRADE) approach was used to assess the certainty of evidence and to draft recommendations (Guyatt et al., 2008), and the guidelines are reported according to the Appraisal of Guidelines Research & Evaluation (AGREE) II tool and Reporting Items for Practice Guideline in Healthcare (RIGHT) Checklist (Brouwers et al., 2016).

### Composition of the guidelines drafting group

The guideline panel was established in 2020 and was composed of 21 panel members, of which the 17 voting members represented the veterinary clinical fields of soft tissue and orthopaedic surgery (FS, YW, SB, JM, MN, SC, DW, JER, TM, AV), internal medicine (FA, TS, LRJ, KS, JG), infectious disease (SW) and veterinary microbiology (EB). The remaining members represented the disciplines of veterinary pharmacology (PLT, LP), veterinary epidemiology (MB) and guidelines methodology in human medicine (FF). The work was chaired by an oversight committee (FA, TS, LRJ, KS, SW), and a methodology taskforce (FA, TS, LRJ, KS, MB, FF) was established as a subset of the group.

### Generation of guidelines content and involvement of veterinary practitioners and pet owners

The prioritised clinical questions, outcomes and content of the guidelines were generated by the guideline panel in an iterative process involving electronic Delphi questionnaires and online meetings. The panel defined the target population as dogs and cats presented for common surgical procedures. Procedures or procedure groups were categorised as clean, clean‐contaminated or contaminated according to the CDC surgical wound classification (SWC) (Garner, 1986; Ortega et al., 2012). Dental and ophthalmic procedures or those classed as dirty (inadequate treatment of traumatic wounds, gross purulence, evident infections) were excluded. The primary and critical outcome for decision‐making was the overall SSI rate including all SSI classes (superficial, deep, organ/space or implant‐associated) (Mangram et al., 1999).

To ensure the relevance of the guidelines and to integrate the perspectives of guideline end‐users, structured interviews were conducted with veterinary practitioners who frequently perform surgical procedures in primary care practice across Europe. The results are described in the systematic review (Sørensen et al., under review). Briefly, respondents were presented with estimated baseline risks of superficial, deep, organ/space or implant‐associated SSIs for different procedures and the potential maximal risk reduction from using SAP. Respondents were asked to specify a risk reduction (calculated as the absolute number per 1000 cats/dogs undergoing that procedure) that would be the tipping point where they would or would not use SAP. Clinical effect thresholds were established following GRADE’s updated guidance of the imprecision domain (Zeng et al., 2022). The threshold for the smallest risk reduction considered important by clinicians (small effect threshold) was generated from the decision tipping point for organ/space or implant‐associated SSI risk reduction, that is the prevention of how many organ/space or implant‐associated SSIs would clinicians consider warrants administration of SAP to all 1000 hypothetical animals. The moderate effect threshold was generated from the decision tipping point for deep SSI risk reductions, and the large effect threshold was generated from the decision tipping point for superficial infections. Thresholds for each procedure group for both peri and post‐operative SAP are listed in Table 1.

**Table 1:** Thresholds applied during the guideline recommendation process

Procedure group	SAP	Thresholds		
		Small effect	Moderate effect	Large effect
Neutering	Peri‐operative AM	50	125	200
	Post‐operative AM	50	100	250
Soft tissue (non‐GI)/urologic	Peri‐operative AM	23	50	200
	Post‐operative AM	30	100	250
Gastrointestinal	Peri‐operative AM	30	40	200
	Post‐operative AM	30	70	250
Orthopaedic (all)	Peri‐operative AM	20	60	100
Orthopaedic (non‐implant)	Post‐operative AM	50	125	200
Orthopaedic (implant)	Post‐operative AM	20	110	200

Threshold numbers are the number of animals out of 1000 procedures/animals that veterinarians deemed would constitute a clinically relevant effect of an intervention (absolute effect size)

### Systematic review and judging the certainty of evidence

The systematic reviews, meta‐analyses (MA) and evidence assessment were conducted by the methodology taskforce and oversight committee (Sørensen et al., under review). The certainty of evidence was assessed for each outcome using the GRADE methodology and was based on the risk of bias, imprecision, indirectness of the evidence, inconsistency of the results and publication bias (Guyatt et al., 2008).

### Generation of recommendations

Recommendations were drafted by the panel in March 2024 during a hybrid ENOVAT drafting group meeting held in Copenhagen, Denmark. Prior to the meeting, panel members were provided with a written summary of the systematic review and meta‐analyses. Panel members were also provided with a narrative summary of the harmful effects of antimicrobial therapy on the canine gastrointestinal residual flora that had been previously prepared for the ENOVAT canine acute diarrhoea guidelines (Jessen et al., 2024) and a summary of the stakeholder interviews (Table 1). Immediately prior to the meeting, panel members attended an introductory course delivered by a methodology expert (FF) from McMaster University on the guideline formation process following the GRADE approach. Drafting of recommendations followed the GRADE Evidence to Decision (EtD) framework, and for each recommendation, the following factors were discussed: quality and certainty of the overall evidence; the balance of desirable and undesirable effects; preferences and values of veterinary practitioners; equity; costs; acceptability and feasibility (Alonso‐Coello et al., 2016). The panel defined consensus as 80% agreement prior to drafting recommendations. Agreement was calculated based on 17 voting members. Confirmatory votes were sought during the manuscript drafting phase in order to ensure agreement levels. Strong recommendations are typically based on moderate or high certainty evidence, but may also be made when lower certainty evidence suggests equivalence between the intervention and comparator and where there are appreciable harms of higher certainty associated with one of the options (e.g. adverse drug effects) (Schünemann et al., 2013). Therefore, the panel could make strong recommendations against the intervention even if only low‐ or very low‐certainty evidence was available since substantive harms are linked to the use of SAP. The evidence informing recommendations is outlined in Table 2. The implications of strong and conditional recommendations are described in Table 3. Human SAP guidelines were also consulted, and their recommendations were taken into consideration as indirect evidence wherever veterinary data was limited, albeit with recognition that human guidelines represent very low certainty of evidence because of indirectness (Box 1).

**Table 2:** Evidence informing strong and conditional recommendations

Strong recommendation
1. The efficacy of treatment (recommendation for) or lack thereof (recommendation against) is informed by moderate to high certainty evidence, OR
2. The lack of efficacy of treatment is informed by low‐ to very low‐certainty evidence, but the harms associated with treatment is informed by moderate to high certainty evidence (recommendation against only)

Conditional recommendation
1. The efficacy of treatment and harms of treatment are informed by very low‐certainty evidence, OR
2. The efficacy of treatment or harms of treatment is associated with moderate or high‐certainty evidence, but there is substantial uncertainty about the preferences and values of different patient groups limiting a strong recommendation

**Table 3:** Implications for strong and conditional recommendations

	Strong recommendation	Conditional recommendation
Animals	Most animals in this situation would benefit from the recommended course of action and only a small proportion would not	Many animals in this situation would benefit from the suggested course of action, but some would not
Clinicians	Most animals should receive the recommended course of action	Evidence is inadequate to make a strong recommendation, and/ or different choices might be appropriate for different animals. Be prepared to help pet owners make a decision that is consistent with their values/preferences
Policy makers	The recommendation can be adapted as policy in most situations	Policy making may require substantial debate and involvement of many stakeholders. Policies are also more likely to vary between regions

Modified from Guyatt et al. (2008) and Schünemann et al. (2013)

Box 1 **A note on terminology**

There are subtle differences in the definitions of the terms antimicrobial and antibiotic, principally relating to their targeted spectrum of activity. The term antimicrobial has been used in this article, although the recommendations refer to antibacterial (antibiotic) activity only.

Antimicrobials include substances that can inhibit or kill a range of microbes, including bacteria, fungi or viruses. Biocides, antiseptics as well as all anti‐infectives (antibacterials, anti‐protozoals, anti‐fungals and anti‐virals) are all considered antimicrobials.

Antibiotics are a class of antimicrobial agents used to treat bacterial infections.

### Evaluation of harms of antimicrobials

Administration of antimicrobials may confer harm to the patient, whether human or animal. Harms to the individual include potentiation of dysbiosis and selection of resistant bacteria, from which a novel antimicrobial‐resistant infection may arise at a later point. In a broader sense, antimicrobial administration is a driving force in the development of antimicrobial resistance, one of the greatest threats to public and animal health (Antimicrobial Resistance Collaborators, 2022; Cassini et al., 2019; O’Neill, 2016). Specifically for veterinary medicine, there is a concern for transmission of resistant bacteria and/or plasmids from animals to humans or the environment. On the basis of a high degree of plausible risk and considering a least harms approach, the following assumptions were adopted for evidence assessment; the harms of post‐operative antimicrobials were considered to be supported by high certainty evidence and harms of IV peri‐operative antimicrobials were considered to be supported by moderate‐certainty evidence.

Reporting of adverse events in the included studies adds evidence to support the harms of individual drugs in specific patient groups.

### Generation of good practice statements (ungraded section)

Good practice statements (Lotfi et al., 2022) covering SSI definitions, drug selection, dose, route and timing of antimicrobial administration were generated by an iterative process involving several Delphi rounds and final approval of considerations by the voting panel members.

### Consultation phase

The guideline was available on the ENOVAT website from 20/01/2025 to 31/05/2025 for public consultation. The public consultation phase was announced by the ENOVAT newsletter, and members from ESGVM, ENOVAT and the European College of Veterinary Surgeons (ECVS) were contacted by email/newsletter and encouraged to participate.

### Definitions

For the purposes of these guidelines, the following definitions were used.

#### Neutering

Surgical removal of testes/ovaries via castration or ovariectomy/ovariohysterectomy.

#### Surgical antimicrobial prophylaxis

The pre‐emptive use of antimicrobial medication to reduce the risk of development of a surgical site infection in a patient where no pre‐existing infection is present at the surgical site.

#### Peri‐operative surgical antimicrobial prophylaxis

Administration of antimicrobials in the 2 hours prior to surgery, with potential redosing intraoperatively and the last dose given not more than 24 hours post‐operatively.

#### Post‐operative surgical antimicrobial prophylaxis

Continuation of antimicrobials beyond 24 hours after the surgical procedure for the express purpose of infection prevention.

#### Surgical site infection (SSI)

An infection occurring following a surgical procedure, that involves the skin/mucous membranes and subcutaneous tissue of the incision (superficial incisional) and/or the soft tissue deep to the skin and subcutaneous tissue (deep incisional) of the incision and/or any part of the anatomy (bones, organs and spaces) that was opened or manipulated during the surgical intervention (organ/space) and may involve implants placed during the intervention (implant‐associated).

## RESULTS

The panel drafted ten strong and eight conditional recommendations. All but one recommendation was informed by low‐ or very low‐certainty evidence concerning the effect of peri‐operative or post‐operative antimicrobials. Fifteen recommendations received 100% agreement and three recommendations received between 80% and 100% agreement.

## RECOMMENDATIONS ON SAP FOR CLEAN, SOFT TISSUE PROCEDURES

### Recommendation 1

In dogs and cats undergoing neutering, we recommend against the use of peri‐operative surgical antimicrobial prophylaxis.

*Strong recommendation, low‐certainty evidence*.

*Level of agreement 100%*.

#### Rationale for recommendation 1

##### Priority value of the recommendation

Neutering is one of the most common procedures performed in companion animal practice (Gates et al., 2020). Despite being a typically short duration, clean surgical procedure that is usually performed in young, healthy animals, routine peri‐operative antimicrobial use was reported in 30% to 40% of neutering procedures in surveys of vets in the UK and Australia (Hardefeldt et al., 2017; Knights et al., 2012) in contrast with national recommendations (BSAVA/SAMSoc, 2024; Hardefeldt et al., 2019).

##### Evidence of therapeutic effect

Peri‐operative antimicrobials do not confer a clinically relevant effect in dogs and cats undergoing neutering. Thirty‐five of 426 dogs (8.2%) receiving peri‐operative antimicrobials developed an SSI compared to 38 of 409 (9.3%) dogs in the control group in a single RCT with predominantly clean surgical procedures of the genitourinary tract (Daude‐Lagravei et al., 2018). The absolute risk reduction (ARR) from peri‐operative SAP was calculated as 11 fewer SSI per 1000 animals treated (95% CI 40 fewer to 34 more animals treated). Summary of findings tables are shown in supporting information (Tables S1 and S2). This difference was below the small effect threshold for a clinically relevant reduction in SSI rate, as defined a priori by veterinary practitioners and was therefore considered trivial. Findings from three observational studies (Brown et al., 1997; de Castro et al., 2022; Stetter et al., 2021) involving 1343 dogs and cats mirrored those from the RCT, with an ARR from peri‐operative SAP calculated as 17 fewer SSI per 1000 animals treated (95% CI 31 fewer to 18 more). The panel also took into consideration the low baseline risk of SSI in dogs and cats undergoing neutering and other clean soft tissue surgery. Uncontrolled prospective studies performed without the use of SAP found SSI rates of 0% following castration in 8 dogs and 8 cats (Al‐Gizawiy et al., 2004), 0.4% following ovariohysterectomy or castration in 1213 dogs and 775 cats (Howe, 1997), and of 0% following ovariectomy in 75 cats (Swaffield et al., 2020), although an SSI rate of 28.6% was reported following laparoscopic ovariohysterectomy in 42 dogs (Bydzovsky et al., 2019). The panel also acknowledged indirect evidence from human medicine. In people, SAP (typically a single dose of peri‐operative intravenous antimicrobial) is recommended for women undergoing elective abdominal hysterectomy (Bratzler et al., 2013) due to a significant risk reduction in post‐operative infection (Ayeleke et al., 2017).

Overall, the evidence was downgraded for risk of bias due to very serious limitations in the study execution of the RCT (Daude‐Lagravei et al., 2018) involving deviations from the intended interventions and missing outcome data. Therefore, the recommendation is based on low‐certainty evidence.

##### The balance between desirable and undesirable effects

Harmful effects associated with SAP could include adverse drug effects (Gosling & Martínez‐Taboada, 2018), negative impacts on the gut microbiota (Rudinsky et al., 2022; Stavroulaki et al., 2023), and the potentiation of antimicrobial resistance (AMR) both in the individual animal via the selection of multi‐drug resistant bacteria that pose an SSI risk (Rubinstein et al., 1994) and in wider society due to the impact of antimicrobial use as a driving force for AMR.

None of the studies included in the systematic review reported adverse effects attributable to antimicrobial administration. However, adverse effects may go undetected, may not be specifically queried or recorded, may not appear to be temporally associated with antimicrobial administration, may be attributed to other medications administered to animals undergoing surgical procedures (e.g. anaesthesia or analgesia) or may be considered part of the presenting pathology for which the procedure was undertaken. Adverse gastrointestinal effects may also be considered common expected impacts and go unrecorded.

When balancing the desirable against undesirable effects of antimicrobials in dogs and cats undergoing neutering, the panel found that the undesirable effects outweighed the desirable effects, which appear to be limited and for which high certainty evidence is lacking.

### Recommendation 2

In dogs and cats undergoing neutering, we strongly recommend against the use of post‐operative surgical antimicrobial prophylaxis.

*Strong recommendation, low‐certainty evidence*.

*Level of agreement 100%*.

#### Rationale for recommendation 2

##### Evidence of therapeutic effect

Post‐operative SAP does not confer a clinically relevant effect in dogs and cats undergoing neutering or other clean soft tissue procedures. Of 244 cats undergoing neutering that received post‐operative cefalexin, 8 cats required wound flushing but no change to the antibiotic regimen compared to 6 of 248 cats in the control group that required wound flushing and antibiotic administration, a surrogate outcome indicating an SSI (Chutipongvivate et al., 2022). The absolute risk reduction from post‐operative SAP was calculated as 28 fewer SSI per 1000 animals treated (95% CI 28 fewer to 5 more). This difference was below the small effect threshold for a clinically relevant reduction in SSI rate, as predefined by veterinary practitioners and was therefore considered trivial. The certainty of evidence was downgraded to low certainty due to serious risk of bias (missing outcome data and lack of clear definition of an SSI) and serious indirectness (the RCT only included cats).

Findings from a single prospective observational study involving 184 dogs undergoing a mixture of soft tissue procedures (Espinel‐Rupérez et al., 2019) resulted in a counterintuitive increased risk of SSI from post‐operative SAP, with the calculated absolute risk being 51 more SSI per 1000 animals treated (95% CI 21 fewer to 296 more). Human guidelines consistently recommend against the continuation of SAP beyond 24 hours post‐procedure (post‐operative SAP) for clean or clean‐contaminated, soft tissue or orthopaedic procedures, including those involving the placement of an implant (Allegranzi et al., 2016; Ban et al., 2017; Berríos‐Torres et al., 2017; Bratzler et al., 2013). Specifically for hysterectomy procedures, there is no evidence supporting SAP continuation beyond 24 hours post‐operatively in human medicine (da Costa & Krauss‐Silva, 2004; Dellinger et al., 1994).

##### The balance between desirable and undesirable effects

Adverse events were not documented following antimicrobial administration in the RCT, although how actively this was monitored is not clearly stated. The panel recognised that the risk of adverse effects, negative impacts on the gut microbiota and potentiation of AMR would likely be greater for orally administered medication, and there would also be additional risks to owners administering the drug (e.g. bites, drug exposure) (Kelly et al., 2021). On the basis of this degree of plausible risk and considering a least harms approach, the panel made a strong recommendation not to use post‐operative SAP in neutering procedures.

### Recommendation 3

In dogs and cats undergoing clean soft tissue surgical procedures other than neutering (e.g. splenectomy, dermal mass removal, exploratory laparotomy, gastropexy), we strongly recommend against the use of peri‐operative surgical antimicrobial prophylaxis.

*Strong recommendation, very low‐certainty evidence*.

*Level of agreement 100%*.

#### Rationale for recommendation 3

##### Priority value of the recommendation

Dermal mass removal and clean elective surgery, including exploratory coeliotomy, are frequently encountered procedures considered among the core competencies anticipated of new graduate veterinarians (Greenfield et al., 2004). Over one in 4 respondents reported always using peri‐operative SAP to remove a 1 cm dermal mass in a survey of UK vets (Knights et al., 2012).

##### Evidence of therapeutic effect

The panel considered the same body of evidence as for recommendation 1, although the vast majority of the clean, soft tissue surgical procedures reported in the only relevant RCT were genital (Daude‐Lagravei et al., 2018). A recent retrospective study reported a single SSI in 66 dogs (1.5%) that underwent splenectomy without peri‐operative SAP (Husi et al., 2023). It is worth noting that this SSI rate is lower than most observational studies reporting outcomes after soft tissue procedures. The certainty of evidence informing recommendation 3 was downgraded for serious indirectness and considered very low certainty.

### Recommendation 4

In dogs and cats undergoing clean soft tissue surgical procedures other than neutering (e.g. splenectomy, dermal mass removal, gastropexy), we strongly recommend against the use of post‐operative surgical antimicrobial prophylaxis.

*Strong recommendation, very low‐certainty evidence*.

*Level of agreement 100%*.

#### Rationale for recommendation 4

##### Evidence of therapeutic effect

No RCTs specifically reported SSI risk with and without post‐operative SAP for dogs and cats undergoing clean, soft tissue surgical procedures other than neutering. The findings from the RCT (Chutipongvivate et al., 2022) considered for recommendation 2 were reviewed, and the evidence was further downgraded to very low certainty due to very serious indirectness. Human guidelines consistently recommend against the continuation of SAP beyond 24 hours post‐procedure for clean procedures (Berríos‐Torres et al., 2017; Bratzler et al., 2013).

## RECOMMENDATIONS ON SAP FOR CLEAN‐CONTAMINATED SOFT TISSUE PROCEDURES

### Recommendation 5

In dogs and cats undergoing clean‐contaminated urological surgical procedures, we conditionally recommend against the use of peri‐operative surgical antimicrobial prophylaxis.

*Conditional recommendation, very low‐certainty evidence*.

*Level of agreement 100%*.

One panel member was uncertain and abstained.

*Remarks*: Urological procedures with suspected active infection should be managed according to recommendations 11 and 12.

#### Rationale for recommendation 5

##### Evidence of therapeutic effect

A subset of 38 dogs and cats from a single RCT (Daude‐Lagravei et al., 2018) underwent clean‐contaminated procedures and were considered for recommendations 5, 7 and 9. Only a single case (placebo group) was classified as urologic surgery (Daude‐Lagravei et al., 2018). Three of 20 dogs (15%) undergoing clean‐contaminated procedures that received peri‐operative antimicrobials developed an SSI compared to 2 of 18 (11.1%) dogs in the control group. The ARR from peri‐operative SAP was calculated as 39 more SSI per 1000 animals treated (95% CI 83 fewer to 688 more). The confidence interval crossed the small and large thresholds for a clinically relevant reduction in SSI rate for urological procedures, as previously defined by veterinary practitioners, and the certainty of evidence was therefore downgraded due to extremely serious imprecision as well as very serious risk of bias.

From the observational studies, 964 dogs and cats were included in the meta‐analysis although only 274 cases represented clean‐contaminated procedures (Turk et al., 2015) as data were unavailable for accurate separation by class. The ARR from peri‐operative SAP was calculated as 26 fewer SSI per 1000 animals treated (95% CI 58 fewer to 65 more). The panel also consulted the evidence in human medicine. The American Urological Association (AUA) Best Practice Statement (BPS) recommends the use of a single dose of periprocedural antimicrobial for patients undergoing Class II/clean‐contaminated genitourinary procedures (Lightner et al., 2020) although all international guidelines lack strong data (Ivan & Sindhwani, 2018).

##### The balance between desirable and undesirable effects

Readers are referred to the prior paragraphs outlining the potential harmful effects from antimicrobial use for SAP in dogs and cats. A conditional recommendation not to use peri‐operative SAP for clean‐contaminated urologic procedures was based on the panel’s view that the risks of organ/space SSI development following the opening of a non‐infected urinary tract (definition of clean‐contaminated) were very low. The recommendation is therefore contingent on the surgeon’s confidence in determining the absence of bacterial infection of the tissue that is being transected or spaces that are being entered. Where the balance of doubt favours an infection, readers are referred to recommendations 11 and 12 for contaminated soft tissue surgical procedures.

### Recommendation 6

In dogs and cats undergoing clean‐contaminated urological surgical procedures, we strongly recommend against the use of post‐operative surgical antimicrobial prophylaxis.

*Strong recommendation, very low‐certainty evidence*.

*Level of agreement 100%*.

#### Rationale for recommendation 6

##### Evidence of therapeutic effect

No RCTs reported outcomes with and without post‐operative SAP for clean‐contaminated procedures. The panel considered evidence from one RCT (Chutipongvivate et al., 2022) and one observational study (Espinel‐Rupérez et al., 2019) as per recommendations 2 and 4. The ARR from post‐operative SAP was calculated for 72 urological procedures from the observational study (Espinel‐Rupérez et al., 2019) as 9 more SSI per 1000 animals treated (95% CI 42 fewer to 356 more). The confidence interval for the ARR crossed both the small (30 per 1000) and large thresholds (250 per 1000) derived from the practitioner surveys, for a clinically relevant reduction in SSI rate (albeit the large threshold is crossed in the opposite direction), prompting the downgrading of the certainty of evidence to very low due to extremely serious imprecision, as well as serious risk of bias and serious indirectness.

##### The balance between desirable and undesirable effects

When balancing the desirable against undesirable effects of post‐operative SAP in dogs undergoing clean‐contaminated surgical procedures, the panel found that undesirable effects outweigh the desirable effects, for which documentation is lacking and made a strong recommendation against the use of post‐operative SAP.

### Recommendation 7

In dogs and cats undergoing clean‐contaminated gastrointestinal surgical procedures, we conditionally recommend the use of peri‐operative surgical antimicrobial prophylaxis.

Remark: The panel recommended against the use of peri‐operative SAP for clean‐contaminated surgery involving the stomach alone (gastrotomy).

*Conditional recommendation, very low‐certainty evidence*.

*Level of agreement 94%*.

The panel reached agreement on a conditional recommendation to use peri‐operative SAP for clean‐contaminated gastrointestinal procedures, although one panel member demurred and voted against using peri‐operative antimicrobials for any clean‐contaminated gastrointestinal procedure.

#### Rationale for recommendation 7

##### Evidence of therapeutic effect

The evidence base for this recommendation was similar to recommendation 5, although no procedures in the RCT were reported to involve the gastrointestinal tract (Daude‐Lagravei et al., 2018). Procedures involving the stomach (gastrotomy, gastrectomy) represent a lower SSI risk due to the lower microbial burden in patients with normal gastric pH (Bratzler et al., 2013; LoCicero & Nichols, 1980; Sjöstedt et al., 1989). Furthermore, there is a lower risk of contamination from gastrointestinal tract contents with gastrotomy compared to enterotomy or enterectomy, as the stomach is more amenable to surgical techniques that reduce the contamination risk (e.g. elevation with stay sutures and isolation with laparotomy swabs). The use of a single dose of periprocedural antimicrobial for patients undergoing specific Class II/clean‐contaminated gastroduodenal procedures is recommended in people (Bratzler et al., 2013).

The evidence was downgraded to very low certainty due to very serious risk of bias and extremely serious imprecision (absolute risk reduction crossed both the small and large effect thresholds derived from the practitioner surveys).

The panel recognised that recommendations could be subdivided according to the involvement of specific sections of the gastrointestinal tract. The panel recommended against the use of peri‐operative SAP for clean‐contaminated surgery involving the stomach alone (gastrotomy).

### Recommendation 8

In dogs and cats undergoing clean‐contaminated gastrointestinal surgical procedures, we strongly recommend against the use of post‐operative surgical antimicrobial prophylaxis.

*Strong recommendation, very low‐certainty evidence*.

*Level of agreement 100%*.

#### Rationale for recommendation 8

##### Evidence of therapeutic effect

No RCTs reported outcomes with and without post‐operative SAP for clean‐contaminated gastrointestinal procedures. The ARR from post‐operative SAP was calculated for 15 gastrointestinal procedures from the observational study (Espinel‐Rupérez et al., 2019) as 167 fewer SSI per 1000 animals treated (95% CI 310 fewer to 950 more). A retrospective study evaluating the value of surgical checklists (Launcelott et al., 2019) reported that 34 of 189 (18%) dogs undergoing surgical removal of a gastrointestinal foreign body that received post‐operative SAP developed an SSI compared to 18 of 113 (15.9%) dogs that did not receive post‐operative SAP. The certainty of evidence was considered very low due to extremely serious imprecision, serious risk of bias and serious indirectness.

### Recommendation 9

In dogs and cats undergoing clean‐contaminated surgical procedures not involving the urological or gastrointestinal tracts, for example corrective interventions for brachycephalic obstructive airway syndrome, we strongly recommend against the use of peri‐operative surgical antimicrobial prophylaxis.

*Strong recommendation, very low‐certainty evidence*.

*Level of agreement 100%*.

The evidence base for this recommendation was similar to recommendations 5 and 7 (Daude‐Lagravei et al., 2018), downgraded for very serious risk of bias and extremely serious imprecision. Current guidelines in human medicine recommend the use of SAP for the majority of clean‐contaminated procedures involving the head and neck (Bratzler et al., 2013), although the procedures and patient populations (head and neck cancer surgery) are quite different from most dogs and cats undergoing surgery involving this region. SAP is not recommended for adenoidectomy, tonsillectomy or septoplasty in people, as SSI rates were not lower in RCTs evaluating SAP for these procedures (Dhiwakar et al., 2012).

### Recommendation 10

In dogs and cats undergoing clean‐contaminated surgical procedures, not involving the urological or gastrointestinal tracts, for example corrective interventions for brachycephalic obstructive airway syndrome, we recommend against the use of post‐operative surgical antimicrobial prophylaxis.

*Strong recommendation, very low‐certainty evidence*.

*Level of agreement 100%*.

#### Rationale for recommendation 10

##### Evidence of therapeutic effect

The evidence base for this recommendation was similar to recommendations 6 and 8, downgraded for serious risk of bias, serious indirectness and extremely serious imprecision.

## RECOMMENDATIONS ON SAP FOR CONTAMINATED SOFT TISSUE PROCEDURES

### Recommendation 11

In dogs and cats undergoing contaminated soft tissue surgical procedures, we conditionally recommend the use of peri‐operative surgical antimicrobial prophylaxis.

*Conditional recommendation, very low‐certainty evidence*.

*Level of agreement 100%*.

#### Rationale for recommendation 11

##### Evidence of therapeutic effect

Contaminated surgical procedures (e.g. cystotomy in an animal with a confirmed bacterial urinary tract infection) likely constitute a minor proportion of the procedures performed in practice and were not represented in any of the randomised controlled antimicrobial treatment trials (Sørensen et al., under review). A subset of 16 dogs undergoing contaminated soft tissue surgical procedures was included in a single observational study (Brown et al., 1997) with no SSIs reported in 2 dogs treated with peri‐operative antimicrobials compared to 4 SSIs in 14 dogs (28.6%) that had not received peri‐operative SAP. The overall certainty of the evidence informing the recommendation is very low, due to a very serious risk of bias and extremely serious imprecision.

##### The balance between desirable and undesirable effects

When balancing the desirable against undesirable effects of antimicrobials in dogs and cats undergoing contaminated soft tissue surgical procedures, the panel found that the potential desirable effects outweigh the undesirable effects and offered a conditional recommendation to use peri‐operative SAP for contaminated soft tissue surgical procedures.

### Recommendation 12

In dogs and cats undergoing contaminated soft tissue surgical procedures (e.g. gastrointestinal spillage), we suggest that post‐operative surgical antimicrobial prophylaxis should be used for 3 to 5 days, with daily review and adaptation/de‐escalation in line with culture and susceptibility test results.

*Conditional recommendation, very low‐certainty evidence*.

*Level of agreement 88%*.

#### Rationale for recommendation 12

##### Evidence of therapeutic effect

No RCTs reported outcomes with and without post‐operative SAP for contaminated procedures. However, gastrointestinal spillage has been associated with increased post‐operative infectious complications in people (Mahajna et al., 2005). The optimal duration of extension is not clear but may be extrapolated from traumatic injuries to the bowel (Dellinger et al., 1994; Heseltine et al., 1986). The 3 to 5 days proposed here is longer than typically recommended in human medicine (48 hours). The certainty of evidence was downgraded for extremely serious indirectness.

##### The balance between desirable and undesirable effects

Since any procedure can be re‐classified as contaminated during the procedure following spillage of the contents of any opened (gastrointestinal or urogenital) viscus or recognition of a previously unidentified acute but non‐purulent inflammation at the surgical site, and since this exposure could increase the risk of an SSI, the panel offered a conditional recommendation for the use of post‐operative SAP for contaminated soft tissue surgical procedures in dogs and cats.

## RECOMMENDATIONS ON SAP FOR ORTHOPAEDIC PROCEDURES

### Recommendation 13

In dogs and cats undergoing clean orthopaedic surgical procedures not involving the placement of an implant, we conditionally recommend against the use of peri‐operative surgical antimicrobial prophylaxis.

*Conditional recommendation, very low‐certainty evidence*.

*Level of agreement 100%*.

#### Priority value of the recommendation

Clean orthopaedic and neurosurgical procedures that do not involve the placement of an implant include arthroscopy, hemilaminectomy, planned implant removal and amputation.

#### Rationale for recommendation 13

##### Evidence of therapeutic effect

Five of 185 dogs (2.7%) undergoing clean orthopaedic procedures that received peri‐operative antimicrobials developed an SSI compared to 7 of 129 (5.4%) dogs in the control group (Holmberg, 1985; Vasseur et al., 1985; Whittem et al., 1999). The absolute risk reduction from peri‐operative SAP was calculated as 34 fewer SSI per 1000 animals treated (95% CI 47 fewer to 7 more). This difference was below the moderate effect threshold (60) for a clinically relevant reduction in SSI rate, as defined a priori by veterinary practitioners and was therefore considered small. Outcome data from 314 dogs and cats from 3 RCTs comparing peri‐operative SAP with placebo were considered for recommendations 13, 15 and 17 (Holmberg, 1985; Vasseur et al., 1985; Whittem et al., 1999). Data from a further RCT (Daude‐Lagravei et al., 2018) which included 252 orthopaedic procedures were excluded from the analysis as the outcomes of 621 soft tissue procedures could not be separated out, leading to unacceptable weighting and potential imprecision. A second RCT (Vasseur et al., 1985) reporting a mixture of procedure types was included as 106 of 128 procedures were orthopaedic. Two observational studies (Brown et al., 1997; Turk et al., 2015) reported outcome data for 1776 dogs and cats comparing peri‐operative SAP with no peri‐operative antimicrobial for a mixture of procedures including orthopaedics. Findings from the observational studies mirrored those from the RCTs, with an absolute risk reduction from peri‐operative SAP calculated as 19 fewer SSI per 1000 animals treated (95% CI 35 fewer to 16 more). Additionally, retrospective studies found SSI rates of 0.6% following hemilaminectomy in 154 dogs (Dyall & Schmökel, 2018), of 1.2% following hemilaminectomy in 83 dogs (Mojarradi et al., 2021) and of 0.3% following partial percutaneous discectomy in 331 dogs (Kinzel et al., 2005); all without the use of SAP. Human guidelines recommend against the use of SAP for orthopaedic procedures without instrumentation or implantation of foreign materials (Bratzler et al., 2013) although this recommendation is based on low‐certainty evidence described as expert opinion or data extrapolated from evidence for general principles and other procedures. The evidence was downgraded for risk of bias due to very serious limitations in the study execution (missing outcome data and measurement of the outcome) and due to serious imprecision (95% CI crossed one effect threshold). Furthermore, the majority of orthopaedic procedures included involved the placement of an implant. Nonetheless, the panel argues that this dataset could be considered without downgrading for indirectness because of the direction of likely bias (implant‐associated procedures are likely at greater risk of SSI, and if there is increased benefit of SAP, the bias would be towards the intervention). Since the effect of the intervention was deemed small, the potential impact of bias is likely small. The recommendation is nonetheless based on very low‐certainty evidence.

##### The balance between desirable and undesirable effects

Taking into consideration the low SSI rates and absence of serious morbidity concerns, the panel contended that routine SAP for orthopaedic procedures not involving the placement of an implant was not justified given the potential for adverse drug effects and the imposition of a selection pressure favouring resistant bacteria.

### Recommendation 14

In dogs and cats undergoing clean orthopaedic surgical procedures not involving the placement of an implant, we strongly recommend against the use of post‐operative surgical antimicrobial prophylaxis.

*Strong recommendation, low‐certainty evidence*.

*Level of agreement 100%*.

#### Rationale for recommendation 14

##### Evidence of therapeutic effect

No dogs and cats undergoing orthopaedic procedures without placement of implants were included in any RCT. A subset of 149 dogs that underwent orthopaedic procedures involving implants other than Tibial Plateau Levelling Osteotomy (TPLOs) (Aiken et al., 2015) was considered for this recommendation. Three of 75 dogs (4.0%) undergoing orthopaedic procedures involving implants other than TPLOs that received post‐operative antimicrobials developed an SSI compared to 2 of 74 (2.7%) dogs in the control group. The absolute risk reduction from post‐operative SAP was calculated as 13 more SSI per 1000 animals treated (95% CI 20 fewer to 205 more). Multiple human guidelines recommend against the continuation of SAP beyond 24 hours post‐procedure for all orthopaedic procedures (Allegranzi et al., 2016; Ban et al., 2017; Berríos‐Torres et al., 2017; Bratzler et al., 2013).

The certainty of evidence was downgraded due to serious risk of bias and serious indirectness. Although the confidence interval crossed the small and large effect thresholds for a clinically relevant increase in SSI rate, further downgrading for imprecision was not applied since the direction of movement (SAP leading to an increased SSI rate) was not mechanistically sound.

##### The balance between desirable and undesirable effects

The panel found that undesirable effects clearly outweighed the desirable effects, for which evidence is lacking. On this basis, the panel made a strong recommendation not to use SAP post‐operatively for orthopaedic surgical procedures not involving the placement of an implant.

### Recommendation 15

In dogs and cats undergoing clean orthopaedic surgical procedures involving the placement of an implant (excluding TPLO), we conditionally recommend the use of peri‐operative surgical antimicrobial prophylaxis.

*Conditional recommendation, very low‐certainty evidence*.

*Level of agreement 100%*.

#### Rationale for recommendation 15

##### Evidence of therapeutic effect

The same evidence was considered as for recommendation 13, and the certainty of evidence was graded similarly. Additionally, a prospective observational study reported an SSI rate of 3.1% following radial or ulnar fracture repair in 25 dogs and 7 cats using bone plate fixation without the use of SAP (Schmökel et al., 2021). Human guidelines recommend the use of SAP for orthopaedic procedures that involve implant material (Bratzler et al., 2013) based on the established efficacy of SAP to reduce SSI rates in fracture procedures (Gillespie & Walenkamp, 2010; Southwell‐Keely et al., 2004).

##### The balance between desirable and undesirable effects

For orthopaedic surgical procedures involving the placement of an implant in dogs and cats, the panel made a conditional recommendation to use SAP peri‐operatively because of the perceived greater risk of SSI and, most importantly, the challenges in treating implant‐associated infections, which may be very costly and require long‐term antimicrobial use or surgical revision.

### Recommendation 16

In dogs and cats undergoing clean orthopaedic surgical procedures involving the placement of an implant (excluding TPLO), we strongly recommend against the use of post‐operative surgical antimicrobial prophylaxis.

*Strong recommendation, moderate‐certainty evidence*.

*Level of agreement 100%*.

#### Rationale for recommendations 16

##### Evidence of therapeutic effect

The same subset of 149 dogs as for recommendation 14 that underwent orthopaedic procedures involving implants other than TPLOs (Aiken et al., 2015) was considered without downgrading the evidence for indirectness since these procedures were directly relevant to the recommendation.

### Recommendation 17

In dogs undergoing a Tibial Plateau Levelling Osteotomy (TPLO) procedure, we conditionally recommend the use of peri‐operative surgical antimicrobial prophylaxis.

*Conditional recommendation, very low‐certainty evidence*.

*Level of agreement 100%*.

#### Priority value of the recommendation

Tibial plateau levelling osteotomy (TPLO) has been described as both the most commonly performed surgery for cranial cruciate ligament disease and the procedure most likely to return dogs to normal function (Beer et al., 2018; Bergh et al., 2014; Nanda & Hans, 2019; von Pfeil et al., 2018), although a recent meta‐analysis found non‐inferiority compared to tibial tuberosity advancement (Wemmers et al., 2022). Increased experience with the procedure contributed to shorter procedure duration (Niida et al., 2024) and reduced complication rates (Bergh & Peirone, 2012); the procedure is undertaken widely in both referral and primary care practices. Although this procedure has been performed occasionally in cats (Bartolomé I Gadea & Coppola, 2024; Mindner et al., 2016), the panel did not offer a recommendation for this species.

##### Evidence of therapeutic effect

The same evidence was considered as for recommendations 13 and 15 and was again downgraded due to very serious risk of bias and serious imprecision. Additionally, a prospective observational study reported an SSI rate of 21.1% in 19 dogs after TPLO without the use of any SAP (Löfqvist et al., 2018).

##### The balance between desirable and undesirable effects

The panel made a conditional recommendation to use SAP peri‐operatively in dogs undergoing a TPLO because of the perceived greater risk of SSI and, most importantly, the challenges in treating implant‐associated infections, which may require long‐term antimicrobials or surgical revision.

### Recommendation 18

In dogs undergoing a Tibial Plateau Levelling Osteotomy (TPLO) procedure, we conditionally recommend against the use of post‐operative surgical antimicrobial prophylaxis.

*Conditional recommendation, very low‐certainty evidence*.

*Level of agreement 94%*.

#### Rationale for recommendation 18

##### Evidence of therapeutic effect

Outcome data from 3 RCTs for 467 dogs that underwent TPLOs were considered for recommendation 18 (Aiken et al., 2015; Pratesi et al., 2015; Spencer & Daye, 2018). Fifteen of 239 dogs (6.3%) undergoing TPLOs that received post‐operative antimicrobials developed an SSI compared to 38 of 228 (16.7%) dogs in the control group. The absolute risk reduction from post‐operative SAP was calculated as 102 fewer SSI per 1000 animals treated (95% CI 143 fewer to 13 more). The confidence interval crossed the small and large effect thresholds for a clinically relevant reduction in SSI rate, as predefined by veterinary practitioners, and the certainty of evidence was downgraded for very serious imprecision. Two prospective observational studies (Andrade et al., 2016; Nazarali et al., 2015) reported outcome data for 253 dogs comparing post‐operative SAP with no post‐operative antimicrobial for TPLOs and tibial tuberosity advancement (TTAs). Seven of 112 dogs (6.3%) undergoing TPLOs that received post‐operative antimicrobials developed an SSI compared to 18 of 141 (12.8%) dogs in the control group. The absolute risk reduction from post‐operative SAP was calculated as 82 fewer SSI per 1000 animals treated (95% CI 107 to 23 fewer). A protective effect from post‐operative SAP was also found in a retrospective analysis of risk factors for SSI development after TPLO (Hagen et al., 2020). However, a recent systematic review (Budsberg et al., 2021) concluded that there was insufficient evidence to support the use of post‐operative SAP for TPLOs. A challenge with the interpretation of studies is the definition (or lack thereof) of “post‐operative.” Studies that classified any antimicrobial use after surgery as “post‐operative” could miscategorise animals that only received antimicrobials within the 24 hours peri‐operative window after surgery. This mixing of case and control groups could bias results, as it is impossible to determine whether any beneficial effects were driven by antimicrobial administration beyond that 24 hours window.

##### The balance between desirable and undesirable effects

For TPLOs in dogs, the panel made a conditional recommendation to not use SAP post‐operatively. SAP should be discontinued at the end of the procedure or within 24 hours of the end of the procedure (considered peri‐operative SAP). The panel further recognised that one of the observational studies (Nazarali et al., 2015) that reported a benefit of post‐operative antimicrobials included some dogs that received antimicrobials for <24 hours (within the peri‐operative window) in their post‐operative antimicrobial group. The conditional recommendation made by the panel is not to continue SAP beyond 24 hours, that is no post‐operative SAP.

## COMMON CONSIDERATIONS FOR RECOMMENDATIONS ON SURGICAL ANTIMICROBIAL PROPHYLAXIS IN DOGS AND CATS

### Feasibility, cost and equity

The recommendations outlined here are feasible and are unlikely to have an important impact on equity or costs. There is a small cost‐saving effect of not administering SAP. However, the costs of antimicrobial therapy vary with the size of the dog and the specific product. In most cases, antimicrobial costs are likely to constitute a relatively minor part of the total cost of the surgical procedure. The antimicrobials typically used for SAP are expected to be readily accessible in situations where there is an ability to perform these surgical procedures. There may be access barriers to specific antimicrobials, but access to one or more appropriate drugs (discussed further below) is expected to be common in most countries.

### Preferences and values of veterinary clinicians performing surgical procedures in practice

The values and preferences of veterinary clinicians demonstrated important uncertainty and variability as evidenced by their answers to the thresholds survey. The wide range of responses for each procedure indicated different individual tolerance of post‐operative complications. Ethnographic studies in human medicine have detected different mindsets between medics and surgeons in their approach to antimicrobial use, with surgeons frequently adopting a more defensive strategy (Charani et al., 2019). These guidelines are designed to counter defensive medicine and support decision‐making around SAP based on available evidence.

### Acceptability and facilitators for implementation

The panel recognises that acceptance of a non‐antimicrobial treatment strategy may vary among practitioners and pet owners. In some regions, guidelines will encounter greater resistance to adoption, and implementation strategies should take into account national values and preferences. Client pressure, perceived or true, may pose a barrier to antimicrobial stewardship (Smith et al., 2018), but would ideally be countered through education and communication rather than unnecessary antimicrobial use (Wright et al., 2024). Interestingly, data from the annual report of the Canine Cruciate registry (https://knowledge.rcvs.org.uk/quality‐improvement/canine‐cruciate‐registry/) indicates that nearly 2/3 of veterinarians performing cruciate repair in the UK did not administer any post‐operative SAP, highlighting the potential acceptability of this recommendation among the target audience.

## RECOMMENDATIONS FOR FUTURE RESEARCH ON SURGICAL ANTIMICROBIAL PROPHYLAXIS IN DOGS AND CATS

The panel recommends SAP efficacy trials of all study designs: comparative RCTs and observational studies for all surgical procedures in cats and dogs as well as non‐comparative studies. Outcomes that are important for decision making should be reported, such as superficial, deep, organ and implant SSI and adverse effects. Further research is warranted to better understand practitioner values and fears around withholding SAP. Since guideline impact is dependent on adherence to the recommendations, recognising the innate barriers to implementation could help improve the messaging to address stakeholder concerns. The panel also recognises the challenges in performing large RCTs in veterinary medicine and encourages the publication of smaller datasets that could be incorporated into systematic reviews and meta‐analyses. Other knowledge gaps to fill include a more refined study of post‐operative antimicrobials, as currently available studies typically group any administration after the procedure as post‐operative, hampering proper assessment of what is truly peri‐ versus post‐operative and prohibiting study of the impact of different post‐operative durations. A recent retrospective study, published after the screening period for the systematic review, investigated the effect of peri‐ and post‐operative SAP in 1060 cats and dogs (Paeckel et al., 2024). No significant difference from post‐operative SAP was identified (Paeckel et al., 2024). However, the identified published literature remains limited and highlights a need for future studies that will help to further refine the next iteration of these recommendations (suggested for 2030).

## GOOD PRACTICE STATEMENTS AROUND THE CORRECT ADMINISTRATION OF SURGICAL ANTIMICROBIAL PROPHYLAXIS

The following considerations on the optimal antimicrobial selection and dosing strategy (both in quantity and frequency of repetition) represent the professional opinion of the panel. The panel has not conducted systematic reviews to inform the statements included in this section, and the guidance provided is ungraded. Attention to surgical asepsis, use of appropriate personal protective equipment and adherence to stringent hospital hygiene measures are highly important to reduce SSI risk. These topics are beyond the scope of this document. Readers are referred to:

FECAVA hygiene guidelines:https://www.fecava.org/wp‐content/uploads/2019/03/FECAVA_Infectioncontrol_2018_LR.pdf.

Ontario Animal Health Network Infection Prevention and Control Best Practices for Small Animal Veterinary Clinics:https://www.amrvetcollective.com/assets/your‐practice/resources/OAHN‐IPC‐Guide‐SB‐Final‐Jan0820_All_tagged‐SUR.pdf.

### Agent selection

Specific recommendations as to the preferred antimicrobial to use for SAP were not made as part of these guidelines. This was because of various factors, including regional variation in drug availability, drug licensing and antimicrobial resistance patterns of the most common SSI pathogens. In general, the preparation chosen for SAP should have reliable activity against staphylococcal species. This can vary somewhat by region, mainly based on the prevalence of beta‐lactamase producing staphylococci. Given the predominance of Gram‐negative bacteria, particularly Enterobacteriaceae, as uropathogens and within the gastrointestinal tract, selection of an antimicrobial for prophylaxis that also includes Gram‐negative activity is recommended for procedures involving the gastrointestinal or urinary tract. Additional anaerobic activity (e.g. inclusion of metronidazole in the SAP protocol) may be indicated for procedures involving entry into the colon.

Cefazolin, a first generation cephalosporin, is the most widely recommended drug for SAP in human medicine (Bratzler et al., 2013) and is a recommended option in dogs and cats where it is available. However, ampicillin may be an effective option in regions where beta‐lactamase producing staphylococci are uncommon. Other potential options include cefuroxime (second generation cephalosporin) and parenteral amoxicillin/clavulanic acid.

### Dosing regimens

The basic concept of SAP is that therapeutic levels should be present at the surgical site during the period of highest infection risk. This starts at the time of the first incision and ends at an ill‐defined point after final closure. To achieve therapeutic levels by the start of the procedure, the drug should be administered intravenously 30 to 60 minutes prior to the anticipated time of first incision (Pelligand et al., 2024). Intramuscular, subcutaneous or oral administration should be avoided unless intravenous administration cannot be performed. Where an alternate route of administration is selected, pharmacokinetics of the drug should be reviewed to determine the optimal timing of administration so that there is confidence that therapeutic levels will be present when required.

Intraoperative dosing is necessary to maintain therapeutic levels for longer procedures or where there was a delay from antimicrobial administration to the start of the procedure. Intraoperative dosing of any time‐dependent antimicrobial, such as beta‐lactams, should be considered every two half‐lives of the drug (Table 4). A recent pharmacokinetic meta‐analysis of five beta‐lactam antibiotics (Pelligand et al., 2024) found that only cefazolin provided adequate peri‐operative antibacterial effect with 2‐hourly administration against *E. coli*. Cefuroxime failed uniformly while ampicillin or amoxicillin may be effective, but only if readministered every 1.5 hours (Pelligand et al., 2024). Concentration‐dependent antimicrobials (fluoroquinolones or aminoglycosides) would not require redosing but should not be administered routinely for SAP considering their higher/more critical EMA classifications.

**Table 4:** Reported half‐lifes of antibiotics commonly used for surgical antimicrobial prophylaxis

Agent	Reported *t*_1/2_ (minutes)		References	
	Dogs	Cats	Dog	Cat
Amoxicillin‐clavulanate	102	75.6	Vegas Cómitre et al. (2021)	Yang et al. (2019)
Ampicillin	58.2	86.3	Monaghan et al. (2021)	Goldstein et al. (1995)
Cefazolin	58	70.8	Cagnardi et al. (2018)	Albarellos et al. (2017)
Cefuroxime	59.4	No data	Albarellos et al. (2016, 2020)	
Metronidazole	268	318	Neff‐Davis et al. (1981)	Sekis et al. (2009)

The exact end of the period of risk is not well defined because there is not an instant and complete physiological seal after the surgical wound is closed. The risk is expected to drop substantially at the time of wound closure and further decrease rapidly in the hours after surgery since a fibrin seal is largely complete within 3 to 6 hours (Burke, 1961). For this reason, antimicrobials may be administered up to 24 hours after the procedure and still be classified as peri‐operative SAP. Continuation of SAP after the time of wound closure is not recommended unless the procedure is classified as contaminated. Interestingly, a systematic review in people found that 60% of SSIs were identified after the patient was discharged from hospital (Woelber et al., 2016). A similar breakdown of the timing of SSI detection has not been performed in veterinary patients. The duration of post‐operative antimicrobial administration for contaminated procedures should be tailored to the particular situation (degree of contamination, source of contamination). A 3 to 5 day course is likely adequate in most cases (expert opinion).

### Prophylaxis approach in patients already receiving antimicrobials

In patients that are already receiving antimicrobials for therapeutic purposes, the drug, route and timing should be reviewed. The goals of therapeutic and SAP use are different, with the latter focusing on maintaining therapeutic levels throughout the entire period of colonisation risk. If the drug that the patient is already being treated with would be an appropriate SAP drug, the drug should be continued, but with a dosing regimen to ensure therapeutic levels at the time of surgery. This may involve specific timing of the procedure, slight alteration in the therapeutic dosing regimen, or administration of additional doses at the time of surgery to meet the SAP goals. If the drug that is being used is not appropriate for SAP or the dosing regimen cannot be adequately managed to proper SAP timing, administration of a recommended SAP drug (e.g. cefazolin) as per any other procedure can be used unless there is evidence of a drug interaction with the therapeutic drug, a highly unlikely situation.

### Intraoperative redosing (Tables 4 and 5)

**Table 5:** Suggested options including dosing for intravenous peri‐operative surgical antimicrobial prophylaxis

Antimicrobial	Dose (mg/kg)	Frequency of redosing
Amoxicillin‐clavulanate	20	q1.5h
Ampicillin	20	q1.5h
Cefazolin	22 to 25	q2h
Cefuroxime	20	q2h
Metronidazole*	15	q8h

* Metronidazole is indicated as an adjunctive agent (to be used alongside one of the antimicrobial options described above) for procedures involving entry to the large intestine only. The doses were collated from different national guidelines included in Pelligand et al. (2024) and Sørensen et al. (2024). The redosing intervals were computed from pharmacokinetic re‐analysis of individual dog raw plasma concentrations for all routinely used molecules and dosage regimens. The aim of this exercise was to propose a common benchmark to support dosage regimens and redosing intervals when surgical antimicrobial prophylaxis was indicated. For more information on the redosing interval according to suspected contaminants (*Staphylococcus* or *E. coli*), see Pelligand et al. (2024)

Intraoperative redosing of *time‐dependent antimicrobial agents* is indicated if the time between the loading dose administration and duration of the procedure exceeds two elimination half‐lives of the antimicrobial agent (Pelligand et al., 2024). Adaptations to the dosing regimen may be warranted in animals considered critically ill as pharmacokinetic properties of amoxicillin‐clavulanate were much more variable in this patient population (Vegas Cómitre et al., 2021).

### Adaptations to SAP according to procedure duration

Given the increased SSI risk with increasing surgical and anaesthesia duration (Espinel‐Rupérez et al., 2019; Eugster et al., 2004; Mayhew et al., 2012; Nicholson et al., 2002; Stetter et al., 2021; Yap et al., 2015), peri‐operative antimicrobials are recommended for any procedure that is scheduled to last longer than 90 minutes. Due to unforeseen circumstances, some surgical procedures may not occur as planned. For example, intraoperative complications can prolong surgical time beyond 90 minutes. However, in such circumstances, the late introduction of antimicrobials will not provide immediate effective action at the surgery site. Nonetheless, given the anticipated increase in SSI risk, where the extension of duration is significant, it seems reasonable to consider antimicrobial administration and manage as per the recommendations for contaminated procedures.

## LIMITATIONS

This study included the views of veterinary practitioners as they were deemed most likely to be making the decisions around SAP administration. However, the perspectives of pet owners and veterinary technicians or nurses were not evaluated. These stakeholders could influence the SAP use decision depending on their degree of tolerance/acceptance of SSIs.

### Author contributions

F.A. and J.S.W. designed and chaired the guidelines process, contributed to the supporting scoping and systematic reviews and drafted the manuscript. T.M.S., L.R.J. and K.S. conducted the supporting systematic review, assisted with the guidelines process and design of stakeholder interviews and contributed to the drafting of the manuscript. F.F. provided technical assistance around the implementation of the GRADE process. All other authors conducted structured interviews with practitioners, contributed to the selection of guideline content and generation of recommendations, reviewed the manuscript and approved the final version.

### Conflict of interest

This guideline document is based upon work from the COST Action European Network for Optimization of Veterinary Antimicrobial Treatment (CA18217), supported by COST (European Cooperation in Science and Technology) (www.cost.eu). The panel members did not declare any substantive conflicts of interest. Many panel members are actively involved in antimicrobial stewardship activities, including formulating national guidelines.

## Funding information

Linnaeus Veterinary Limited supported the costs of the Open Access Publication Charges.

## Supporting information


**Data S1.** Response to comments during consultation phase

**Table S1.** Summary of findings for all PICOs relating to peri‐operative SAP compared to no SAP in dogs and cats

**Table S2.** Summary of findings for all PICOs relating to post‐operative SAP compared to no SAP in dogs and cats

## Data Availability

The data that support the findings of this study are available from the corresponding author upon reasonable request.

## References

[jsap70072-bib-0001] Aiken, M.J. , Hughes, T.K. , Abercromby, R.H. , Holmes, M.A. & Anderson, A.A. (2015) Prospective, randomized comparison of the effect of two antimicrobial regimes on surgical site infection rate in dogs undergoing orthopedic implant surgery. Veterinary Surgery, 44, 661–667. 25780942 10.1111/vsu.12327

[jsap70072-bib-0002] Albarellos, G.A. , Montoya, L. , Lorenzini, P.M. , Passini, S.M. , Lupi, M.P. & Landoni, M.F. (2016) Pharmacokinetics of cefuroxime after intravenous, intramuscular, and subcutaneous administration to dogs. Journal of Veterinary Pharmacology and Therapeutics, 39, 40–44. 25982523 10.1111/jvp.12239

[jsap70072-bib-0003] Albarellos, G.A. , Montoya, L. , Passini, S.M. , Lupi, M.P. , Lorenzini, P.M. & Landoni, M.F. (2017) Cefazolin pharmacokinetics in cats under surgical conditions. Journal of Feline Medicine and Surgery, 19, 992–997. 27609113 10.1177/1098612X16666594 PMC11110984

[jsap70072-bib-0004] Albarellos, G.A. , Passini, S.M. , Lupi, M.P. , Aramayona, S. , Lorenzini, P.M. , Montoya, L. et al. (2020) Effect of food on the pharmacokinetics of oral cefuroxime axetil in dogs. Journal of Veterinary Pharmacology and Therapeutics, 43, 297–302. 32157713 10.1111/jvp.12854

[jsap70072-bib-0005] Al‐Gizawiy, M. , Ijaz, A. & Khamas, W.A. (2004) A comparison of two castration methods in tomcats and dogs. Pakistan Veterinary Journal, 24, 134–138.

[jsap70072-bib-0006] Allegranzi, B. , Zayed, B. , Bischoff, P. , Kubilay, N.Z. , de Jonge, S. , de Vries, F. et al. (2016) New WHO recommendations on intraoperative and postoperative measures for surgical site infection prevention: an evidence‐based global perspective. Lancet Infectious Diseases, 16, e288–e303. 27816414 10.1016/S1473-3099(16)30402-9

[jsap70072-bib-0007] Allerton, F. , Prior, C. , Bagcigil, A. , Broens, E. , Callens, B. , Damborg, P. et al. (2021) Overview and evaluation of existing guidelines for rational antimicrobial use in small‐animal veterinary practice in Europe. Antibiotics, 10, 409. 33918617 10.3390/antibiotics10040409 PMC8069046

[jsap70072-bib-0008] Alonso‐Coello, P. , Oxman, A.D. , Moberg, J. , Brignardello‐Petersen, R. , Akl, E.A. , Davoli, M. et al. (2016) GRADE evidence to decision (EtD) frameworks: a systematic and transparent approach to making well informed healthcare choices. 2: clinical practice guidelines. BMJ, 353, i2089. 27365494 10.1136/bmj.i2089

[jsap70072-bib-0009] Andrade, N. , Schmiedt, C.W. , Cornell, K. , Radlinsky, M.G. , Heidingsfelder, L. , Clarke, K. et al. (2016) Survey of intraoperative bacterial contamination in dogs undergoing elective orthopedic surgery. Veterinary Surgery, 45, 214–222. 26757033 10.1111/vsu.12438

[jsap70072-bib-0010] Antimicrobial Resistance Collaborators . (2022) Global burden of bacterial antimicrobial resistance in 2019: a systematic analysis. Lancet, 399, 629–655. 37500506 10.1136/ebnurs-2022-103540

[jsap70072-bib-0011] Ayeleke, R.O. , Mourad, S. , Marjoribanks, J. , Calis, K.A. & Jordan, V. (2017) Antibiotic prophylaxis for elective hysterectomy. Cochrane Database of Systematic Reviews, 6, CD004637. 28625021 10.1002/14651858.CD004637.pub2 PMC6441670

[jsap70072-bib-0012] Ban, K.A. , Minei, J.P. , Laronga, C. , Harbrecht, B.G. , Jensen, E.H. , Fry, D.E. et al. (2017) American College of Surgeons and Surgical Infection Society: surgical site infection guidelines, 2016 update. Journal of the American College of Surgeons, 224, 59–74. 27915053 10.1016/j.jamcollsurg.2016.10.029

[jsap70072-bib-0013] Bartolomé I Gadea, P. & Coppola, M. (2024) Combination of TPLO, medial and lateral augmentation techniques for the treatment of traumatic stifle luxation in a cat. JFMS Open Reports, 10, 20551169241247439. 38784085 10.1177/20551169241247439 PMC11113046

[jsap70072-bib-0014] Beer, P. , Bockstahler, B. & Schnabl‐Feichter, E. (2018) Tibial plateau leveling osteotomy and tibial tuberosity advancement – a systematic review. Tierarztliche Praxis Ausgabe K Kleintiere Heimtiere, 46, 223–235. 30149404 10.15654/TPK-170486

[jsap70072-bib-0015] Bergh, M.S. & Peirone, B. (2012) Complications of tibial plateau levelling osteotomy in dogs. Veterinary and Comparative Orthopaedics and Traumatology, 25, 349–358. 22534675 10.3415/VCOT-11-09-0122

[jsap70072-bib-0016] Bergh, M.S. , Sullivan, C. , Ferrell, C.L. , Troy, J. & Budsberg, S.C. (2014) Systematic review of surgical treatments for cranial cruciate ligament disease in dogs. Journal of the American Animal Hospital Association, 50, 315–321. 25028440 10.5326/JAAHA-MS-6356

[jsap70072-bib-0017] Berríos‐Torres, S.I. , Umscheid, C.A. , Bratzler, D.W. , Leas, B. , Stone, E.C. , Kelz, R.R. et al. (2017) Centers for Disease Control and Prevention guideline for the prevention of surgical site infection, 2017. JAMA Surgery, 152, 784–791. 28467526 10.1001/jamasurg.2017.0904

[jsap70072-bib-0018] Bratzler, D.W. , Dellinger, E.P. , Olsen, K.M. , Perl, T.M. , Auwaerter, P.G. , Bolon, M.K. et al. (2013) Clinical practice guidelines for antimicrobial prophylaxis in surgery. Surgical Infections, 14, 73–156. 23461695 10.1089/sur.2013.9999

[jsap70072-bib-0019] Brouwers, M.C. , Kerkvliet, K. , Spithoff, K. & AGREE Next Steps Consortium . (2016) The AGREE reporting checklist: a tool to improve reporting of clinical practice guidelines. BMJ, 352, i1152. 26957104 10.1136/bmj.i1152 PMC5118873

[jsap70072-bib-0020] Brown, D.C. , Conzemius, M.G. , Shofer, F. & Swann, H. (1997) Epidemiologic evaluation of postoperative wound infections in dogs and cats. Journal of the American Veterinary Medical Association, 210, 1302–1306. 9143534

[jsap70072-bib-0021] BSAVA/SAMSoc . (2024) Protect me [WWW document]. Available from: https://www.bsavalibrary.com/content/chapter/10.22233/9781913859312.chap4_1#supplementary_data [Accessed 28th December 2024].

[jsap70072-bib-0022] Budsberg, S.C. , Torres, B.T. & Sandberg, G.S. (2021) Efficacy of postoperative antibiotic use after tibial plateau leveling osteotomy in dogs: a systematic review. Veterinary Surgery, 50, 729–739. 33709459 10.1111/vsu.13603

[jsap70072-bib-0023] Burke, J.F. (1961) The effective period of preventive antibiotic action in experimental incisions and dermal lesions. Surgery, 50, 161–168. 16722001

[jsap70072-bib-0024] Bydzovsky, N.D. , Bockstahler, B. & Dupré, G. (2019) Single‐port laparoscopic‐assisted ovariohysterectomy with a modified glove‐port technique in dogs. Veterinary Surgery, 48, 715–725. 31161631 10.1111/vsu.13242 PMC6618065

[jsap70072-bib-0025] Cagnardi, P. , Di Cesare, F. , Toutain, P.‐L. , Bousquet‐Mélou, A. , Ravasio, G. & Villa, R. (2018) Population pharmacokinetic study of cefazolin used prophylactically in canine surgery for susceptibility testing breakpoint determination. Frontiers in Pharmacology, 9, 1137. 30356800 10.3389/fphar.2018.01137 PMC6190795

[jsap70072-bib-0026] Cassini, A. , Högberg, L.D. , Plachouras, D. , Quattrocchi, A. , Hoxha, A. , Simonsen, G.S. et al. (2019) Attributable deaths and disability‐adjusted life‐years caused by infections with antibiotic‐resistant bacteria in the EU and the European economic area in 2015: a population‐level modelling analysis. The Lancet Infectious Diseases, 19, 56–66. 30409683 10.1016/S1473-3099(18)30605-4 PMC6300481

[jsap70072-bib-0027] Charani, E. , Ahmad, R. , Rawson, T.M. , Castro‐Sanchèz, E. , Tarrant, C. & Holmes, A.H. (2019) The differences in antibiotic decision‐making between acute surgical and acute medical teams: an ethnographic study of culture and team dynamics. Clinical Infectious Diseases, 69, 12–20. 30445453 10.1093/cid/ciy844 PMC6579961

[jsap70072-bib-0028] Chutipongvivate, P. , Punyapornwithaya, V. , Thongkorn, K. & Lampang, K.N. (2022) Incidence of short‐term complications after sterilization between pre‐operative antibiotic versus pre‐ and post‐operative antibiotic administration in female cats. Veterinary Integrative Sciences, 20, 379–389.

[jsap70072-bib-0029] da Costa, R.J.M. & Krauss‐Silva, L. (2004) Systematic review and meta‐analysis of antibiotic prophylaxis in abdominal hysterectomy. Cadernos de Saúde Pública, 20, S175–S189. 15608932 10.1590/s0102-311x2004000800013

[jsap70072-bib-0030] Daude‐Lagravei, A. , Carozzo, C. , Fayolle, P. , Yiguier, E. , Viateau, Y. & Moissonnier, P. (2018) Infection rates in surgical procedures: a comparison of cefalexin vs. a placebo. Veterinary and Comparative Orthopaedics and Traumatology, 14, 146–150.

[jsap70072-bib-0031] de Castro, A.G.R. , Magalhães, C.d.S. , Sala, P.L. , Borges, T.B. , Zaniolo, M.M. , Tramontin, R.S. et al. (2022) Auto‐hemoterapia em orquiectomia de cães. Ciência Animal (Impresso), 32, 17–27.

[jsap70072-bib-0032] Dellinger, E.P. , Gross, P.A. , Barrett, T.L. , Krause, P.J. , Martone, W.J. , McGowan, J.E. et al. (1994) Quality standard for antimicrobial prophylaxis in surgical procedures Infectious Diseases Society of America. Clinical Infectious Diseases, 18, 422–427. 8011827 10.1093/clinids/18.3.422

[jsap70072-bib-0033] Dhiwakar, M. , Clement, W.A. , Supriya, M. & McKerrow, W. (2012) Antibiotics to reduce post‐tonsillectomy morbidity. Cochrane Database of Systematic Reviews, 12, CD005607. 18425926 10.1002/14651858.CD005607.pub2

[jsap70072-bib-0034] Dyall, B.A.R. & Schmökel, H.G. (2018) Surgical site infection rate after hemilaminectomy and laminectomy in dogs without perioperative antibiotic therapy. Veterinary and Comparative Orthopaedics and Traumatology, 31, 202–213. 29679951 10.1055/s-0038-1639365

[jsap70072-bib-0035] Espinel‐Rupérez, J. , Martín‐Ríos, M.D. , Salazar, V. , Baquero‐Artigao, M.R. & Ortiz‐Díez, G. (2019) Incidence of surgical site infection in dogs undergoing soft tissue surgery: risk factors and economic impact. Veterinary Record Open, 6, e000233. 31673370 10.1136/vetreco-2017-000233 PMC6802975

[jsap70072-bib-0036] Eugster, S. , Schawalder, P. , Gaschen, F. & Boerlin, P. (2004) A prospective study of postoperative surgical site infections in dogs and cats. Veterinary Surgery, 33, 542–550. 15362994 10.1111/j.1532-950X.2004.04076.x

[jsap70072-bib-0037] Garner, J.S. (1986) CDC guideline for prevention of surgical wound infections, 1985. Supersedes guideline for prevention of surgical wound infections published in 1982. (originally published in November 1985). Revised. Infection Control, 7, 193–200. 3633903 10.1017/s0195941700064080

[jsap70072-bib-0038] Gates, M.C. , Littlewood, K.E. , Kongara, K. , Odom, T.F. & Sawicki, R.K. (2020) Experience of practicing veterinarians with supervising final‐year students and new graduates in performing desexing surgeries. Journal of Veterinary Medical Education, 47, 465–474. 32412365 10.3138/jvme.0918-100r

[jsap70072-bib-0039] Gillespie, W.J. & Walenkamp, G.H. (2010) Antibiotic prophylaxis for surgery for proximal femoral and other closed long bone fractures. Cochrane Database of Systematic Reviews, 2010, CD000244. 11279687 10.1002/14651858.CD000244

[jsap70072-bib-0040] Goldstein, R. , Lavy, E. , Shem‐Tov, M. , Glickman, A. , Bark, H. & Ziv, G. (1995) Pharmacokinetics of ampicillin administered intravenously and intraosseously to kittens. Research in Veterinary Science, 59, 186–187. 8525114 10.1016/0034-5288(95)90059-4

[jsap70072-bib-0041] Gosling, M.J. & Martínez‐Taboada, F. (2018) Adverse reactions to two intravenous antibiotics (Augmentin and Zinacef) used for surgical prophylaxis in dogs. Veterinary Record, 182, 80. 29051310 10.1136/vr.104496

[jsap70072-bib-0042] Greenfield, C.L. , Johnson, A.L. & Schaeffer, D.J. (2004) Frequency of use of various procedures, skills, and areas of knowledge among veterinarians in private small animal exclusive or predominant practice and proficiency expected of new veterinary school graduates. Journal of the American Veterinary Medical Association, 224, 1780–1787. 15198262 10.2460/javma.2004.224.1780

[jsap70072-bib-0043] Guyatt, G.H. , Oxman, A.D. , Vist, G.E. , Kunz, R. , Falck‐Ytter, Y. , Alonso‐Coello, P. et al. (2008) GRADE: an emerging consensus on rating quality of evidence and strength of recommendations. BMJ, 336, 924–926. 18436948 10.1136/bmj.39489.470347.AD PMC2335261

[jsap70072-bib-0044] Hagen, C.R.M. , Singh, A. , Weese, J.S. , Marshall, Q. , Linden, A.Z. & Gibson, T.W.G. (2020) Contributing factors to surgical site infection after tibial plateau leveling osteotomy: a follow‐up retrospective study. Veterinary Surgery, 49, 930–939. 32359005 10.1111/vsu.13436

[jsap70072-bib-0045] Hardefeldt, L.Y. , Browning, G.F. , Thursky, K. , Gilkerson, J.R. , Billman‐Jacobe, H. , Stevenson, M.A. et al. (2017) Antimicrobials used for surgical prophylaxis by companion animal veterinarians in Australia. Veterinary Microbiology, 203, 301–307. 28619161 10.1016/j.vetmic.2017.03.027

[jsap70072-bib-0046] Hardefeldt, L.Y. , Crabb, H.K. , Bailey, K.E. , Johnstone, T. , Gilkerson, J.R. , Billman‐Jacobe, H. et al. (2019) Appraisal of the Australian veterinary prescribing guidelines for antimicrobial prophylaxis for surgery in dogs and cats. Australian Veterinary Journal, 97, 316–322. 31286484 10.1111/avj.12848

[jsap70072-bib-0047] Hassan, S. , Chan, V. , Stevens, J. & Stupans, I. (2021) Factors that influence adherence to surgical antimicrobial prophylaxis (SAP) guidelines: a systematic review. Systematic Reviews, 10, 29. 33453730 10.1186/s13643-021-01577-w PMC7811740

[jsap70072-bib-0048] Heseltine, P.N. , Berne, T.V. , Yellin, A.E. , Gill, M.A. & Appleman, M.D. (1986) The efficacy of cefoxitin vs. clindamycin/gentamicin in surgically treated stab wounds of the bowel. The Journal of Trauma, 26, 241–245. 3951003 10.1097/00005373-198603000-00005

[jsap70072-bib-0049] Holmberg, D.L. (1985) The use of prophylactic penicillin in orthopedic surgery: a clinical trial. Veterinary Surgery, 14, 160–165.

[jsap70072-bib-0050] Howe, L.M. (1997) Short‐term results and complications of prepubertal gonadectomy in cats and dogs. Journal of the American Veterinary Medical Association, 211, 57–62. 9215412

[jsap70072-bib-0051] Hsieh, E.S. , Bollig, E.R. , Beaudoin, A.L. , Morrow, A. & Granick, J.L. (2022) Serial point‐prevalence surveys to estimate antibiotic use in a small animal veterinary teaching hospital, November 2018 to October 2019. Journal of Veterinary Internal Medicine, 36, 244–252. 34773289 10.1111/jvim.16314 PMC8783336

[jsap70072-bib-0052] Husi, B.A. , Arnaldi, L. , Roitner, M. & Nolff, M.C. (2023) Retrospective evaluation of surgical site infection after open splenectomies with and without perioperative prophylactic antibiotic coverage. Tierarztliche Praxis Ausgabe K Kleintiere Heimtiere, 51, 154–160. 37567177 10.1055/a-2105-3593

[jsap70072-bib-0053] Ivan, S.J. & Sindhwani, P. (2018) Comparison of guideline recommendations for antimicrobial prophylaxis in urologic procedures: variability, lack of consensus, and contradictions. International Urology and Nephrology, 50, 1923–1937. 30145652 10.1007/s11255-018-1971-1

[jsap70072-bib-0054] Jessen, L.R. , Werner, M. , Singleton, D. , Prior, C. , Foroutan, F. , Ferran, A.A. et al. (2024) European Network for Optimization of Veterinary Antimicrobial Therapy (ENOVAT) guidelines for antimicrobial use in canine acute diarrhoea. The Veterinary Journal, 307, 106208. 39074542 10.1016/j.tvjl.2024.106208

[jsap70072-bib-0055] Kelly, S.A. , Nzakizwanayo, J. , Rodgers, A.M. , Zhao, L. , Weiser, R. , Tekko, I.A. et al. (2021) Antibiotic therapy and the gut microbiome: investigating the effect of delivery route on gut pathogens. ACS Infectious Diseases, 7, 1283–1296. 33843198 10.1021/acsinfecdis.1c00081 PMC7618120

[jsap70072-bib-0056] Kinzel, S. , Wolff, M. , Buecker, A. , Krombach, G.A. , Stopinski, T. , Afify, M. et al. (2005) Partial percutaneous discectomy for treatment of thoracolumbar disc protrusion: retrospective study of 331 dogs. The Journal of Small Animal Practice, 46, 479–484. 16245661 10.1111/j.1748-5827.2005.tb00276.x

[jsap70072-bib-0057] Knights, C.B. , Mateus, A. & Baines, S.J. (2012) Current British veterinary attitudes to the use of perioperative antimicrobials in small animal surgery. The Veterinary Record, 170, 646. 22562102 10.1136/vr.100292

[jsap70072-bib-0058] Launcelott, Z.A. , Lustgarten, J. , Sung, J. , Samuels, S. , Davis, S. & Davis, G.J. (2019) Effects of a surgical checklist on decreasing incisional infections following foreign body removal from the gastrointestinal tract in dogs. Canadian Veterinary Journal, 60, 67–72. PMC6294029 30651653

[jsap70072-bib-0059] Lightner, D.J. , Wymer, K. , Sanchez, J. & Kavoussi, L. (2020) Best practice statement on urologic procedures and antimicrobial prophylaxis. The Journal of Urology, 203, 351–356. 31441676 10.1097/JU.0000000000000509

[jsap70072-bib-0060] Liu, Z. , Dumville, J.C. , Norman, G. , Westby, M.J. , Blazeby, J. , McFarlane, E. et al. (2018) Intraoperative interventions for preventing surgical site infection: an overview of cochrane reviews. Cochrane Database of Systematic Reviews, 2018, CD012653. 10.1002/14651858.CD012653.pub2 PMC6491077 29406579

[jsap70072-bib-0061] LoCicero, J. & Nichols, R.L. (1980) Sepsis after gastroduodenal operations: relationship to gastric acid, motility, and endogenous microflora. Southern Medical Journal, 73, 878–880. 7384847

[jsap70072-bib-0062] Löfqvist, K. , Kjelgaard‐Hansen, M. & Nielsen, M.B.M. (2018) Usefulness of C‐reactive protein and serum amyloid A in early detection of postoperative infectious complications to tibial plateau leveling osteotomy in dogs. Acta Veterinaria Scandinavica, 60, 30. 29784055 10.1186/s13028-018-0385-5 PMC5963089

[jsap70072-bib-0063] Lotfi, T. , Hajizadeh, A. , Moja, L. , Akl, E.A. , Piggott, T. , Kredo, T. et al. (2022) A taxonomy and framework for identifying and developing actionable statements in guidelines suggests avoiding informal recommendations. Journal of Clinical Epidemiology, 141, 161–171. 34562579 10.1016/j.jclinepi.2021.09.028

[jsap70072-bib-0064] Mahajna, A. , Krausz, M. , Rosin, D. , Shabtai, M. , Hershko, D. , Ayalon, A. et al. (2005) Bowel preparation is associated with spillage of bowel contents in colorectal surgery. Diseases of the Colon and Rectum, 48, 1626–1631. 15981063 10.1007/s10350-005-0073-1

[jsap70072-bib-0065] Mangram, A.J. , Horan, T.C. , Pearson, M.L. , Silver, L.C. & Jarvis, W.R. (1999) Guideline for prevention of surgical site infection, 1999. Centers for Disease Control and Prevention (CDC) hospital infection control practices advisory committee. American Journal of Infection Control, 27, 97–132; quiz 133–134; discussion 96. 10196487

[jsap70072-bib-0066] Mayhew, P.D. , Freeman, L. , Kwan, T. & Brown, D.C. (2012) Comparison of surgical site infection rates in clean and clean‐contaminated wounds in dogs and cats after minimally invasive versus open surgery: 179 cases (2007–2008). Journal of the American Veterinary Medical Association, 240, 193–198. 22217028 10.2460/javma.240.2.193

[jsap70072-bib-0067] Mindner, J.K. , Bielecki, M.J. , Scharvogel, S. & Meiler, D. (2016) Tibial plateau levelling osteotomy in eleven cats with cranial cruciate ligament rupture. Veterinary and Comparative Orthopaedics and Traumatology, 29, 528–535. 27709223 10.3415/VCOT-15-11-0184

[jsap70072-bib-0068] Mojarradi, A. , De Decker, S. , Bäckström, C. & Bergknut, N. (2021) Safety of early postoperative hydrotherapy in dogs undergoing thoracolumbar hemilaminectomy. The Journal of Small Animal Practice, 62, 1062–1069. 34423457 10.1111/jsap.13412

[jsap70072-bib-0069] Monaghan, K.N. , Labato, M.A. & Papich, M.G. (2021) Ampicillin pharmacokinetics in azotemic and healthy dogs. Journal of Veterinary Internal Medicine, 35, 987–992. 33474795 10.1111/jvim.16026 PMC7995374

[jsap70072-bib-0070] Nanda, A. & Hans, E.C. (2019) Tibial plateau leveling osteotomy for cranial cruciate ligament rupture in canines: patient selection and reported outcomes. Veterinary Medicine, 10, 249–255. 31921614 10.2147/VMRR.S204321 PMC6938195

[jsap70072-bib-0071] Nazarali, A. , Singh, A. , Moens, N.M.M. , Gatineau, M. , Sereda, C. , Fowler, D. et al. (2015) Association between methicillin‐resistant staphylococcus pseudintermedius carriage and the development of surgical site infections following tibial plateau leveling osteotomy in dogs. Journal of the American Veterinary Medical Association, 247, 909–916. 26421403 10.2460/javma.247.8.909

[jsap70072-bib-0072] Neff‐Davis, C.A. , Davis, L.E. & Gillette, E.L. (1981) Metronidazole: a method for its determination in biological fluids and its disposition kinetics in the dog. Journal of Veterinary Pharmacology and Therapeutics, 4, 121–127. 7349324 10.1111/j.1365-2885.1981.tb00720.x

[jsap70072-bib-0073] Nicholson, M. , Beal, M. , Shofer, F. & Brown, D.C. (2002) Epidemiologic evaluation of postoperative wound infection in clean‐contaminated wounds: a retrospective study of 239 dogs and cats. Veterinary Surgery, 31, 577–581. 12415527 10.1053/jvet.2002.34661

[jsap70072-bib-0074] Niida, A. , Chou, P.‐Y. , Filliquist, B. , Marcellin‐Little, D.J. , Kapatkin, A.S. & Kass, P.H. (2024) The impact of surgery resident training on the duration of tibial plateau leveling osteotomy surgery. Veterinary Surgery, 53, 808–815. 38764197 10.1111/vsu.14113

[jsap70072-bib-0075] O'Neill J. (2016) Tackling drug‐resistant infections globally: final report and recommendations. May, 2016. Accessed December 2025. https://amr‐review.org/sites/default/files/160518_Final%20paper_with%20cover.pdf.

[jsap70072-bib-0076] Ortega, G. , Rhee, D.S. , Papandria, D.J. , Yang, J. , Ibrahim, A.M. , Shore, A.D. et al. (2012) An evaluation of surgical site infections by wound classification system using the ACS‐NSQIP. The Journal of Surgical Research, 174, 33–38. 21962737 10.1016/j.jss.2011.05.056

[jsap70072-bib-0077] Paeckel, N. , Zablotski, Y. & Meyer‐Lindenberg, A. (2024) The effect of peri‐ and postoperative antibiotic prophylaxis on surgical site infection in surgeries with elective antibiotic administration. Veterinary Journal, 308, 106267. 39549932 10.1016/j.tvjl.2024.106267

[jsap70072-bib-0078] Pelligand, L. , Møller Sørensen, T. , Cagnardi, P. , Toutain, P.‐L. & Allerton, F. (2024) Population pharmacokinetic meta‐analysis of five beta‐lactams antibiotics to support dosing regimens in dogs for surgical antimicrobial prophylaxis. Veterinary Journal, 305, 106136. 38759725 10.1016/j.tvjl.2024.106136

[jsap70072-bib-0079] Pratesi, A. , Moores, A.P. , Downes, C. , Grierson, J. & Maddox, T.W. (2015) Efficacy of postoperative antimicrobial use for clean orthopedic implant surgery in dogs: a prospective randomized study in 100 consecutive cases. Veterinary Surgery, 44, 653–660. 25756814 10.1111/vsu.12326

[jsap70072-bib-0080] Rubinstein, E. , Findler, G. , Amit, P. & Shaked, I. (1994) Perioperative prophylactic cephazolin in spinal surgery. A double‐blind placebo‐controlled trial. Journal of Bone and Joint Surgery British Volume, 76, 99–102. 8300691

[jsap70072-bib-0081] Rudinsky, A.J. , Parker, V.J. , Winston, J. , Cooper, E. , Mathie, T. , Howard, J.P. et al. (2022) Randomized controlled trial demonstrates nutritional management is superior to metronidazole for treatment of acute colitis in dogs. Journal of the American Veterinary Medical Association, 260, S23–S32. 36191142 10.2460/javma.22.08.0349

[jsap70072-bib-0082] Sartelli, M. , Coccolini, F. , Labricciosa, F.M. , Al Omari, A.K.H. , Bains, L. , Baraket, O. et al. (2024) Surgical antibiotic prophylaxis: a proposal for a global evidence‐based bundle. Antibiotics, 13, 100. 38275329 10.3390/antibiotics13010100 PMC10812782

[jsap70072-bib-0083] Schmökel, H. , Skytte, D. & Barsch, M. (2021) Infection rate treating radial and ulnar fractures using bone plate fixation without antibiotic prophylaxis. The Journal of Small Animal Practice, 62, 1079–1084. 34410009 10.1111/jsap.13407

[jsap70072-bib-0084] Schünemann, H.B.J. , Guyatt, G. & Oxman, A. (2013) 6.3.2 confidence in best estimates of magnitude of effects (quality of evidence). In: *Grade handbook for grading quality of evidence and strength of recommendations*.

[jsap70072-bib-0085] Sekis, I. , Ramstead, K. , Rishniw, M. , Schwark, W.S. , McDonough, S.P. , Goldstein, R.E. et al. (2009) Single‐dose pharmacokinetics and genotoxicity of metronidazole in cats. Journal of Feline Medicine and Surgery, 11, 60–68. 19155181 10.1016/j.jfms.2008.06.011 PMC10832785

[jsap70072-bib-0086] Sjöstedt, S. , Levin, P. , Malmborg, A.S. , Bergman, U. & Kager, L. (1989) Septic complications in relation to factors influencing the gastric microflora in patients undergoing gastric surgery. The Journal of Hospital Infection, 13, 191–197. 2567313 10.1016/0195-6701(89)90027-3

[jsap70072-bib-0087] Smith, M. , King, C. , Davis, M. , Dickson, A. , Park, J. , Smith, F. et al. (2018) Pet owner and vet interactions: exploring the drivers of AMR. Antimicrobial Resistance and Infection Control, 7, 46. 29619213 10.1186/s13756-018-0341-1 PMC5879597

[jsap70072-bib-0088] Sørensen, T.M. , Scahill, K. , Ruperez, J.E. , Olejnik, M. , Swinbourne, F. , Verwilghen, D.R. et al. (2024) Antimicrobial prophylaxis in companion animal surgery: a scoping review for European network for optimization of antimicrobial therapy (ENOVAT) guidelines. The Veterinary Journal, 304, 106101. 38490359 10.1016/j.tvjl.2024.106101

[jsap70072-bib-0089] Southwell‐Keely, J.P. , Russo, R.R. , March, L. , Cumming, R. , Cameron, I. & Brnabic, A.J. (2004) Antibiotic prophylaxis in hip fracture surgery: a meta analysis. Clinical Orthopaedics and Related Research, 419, 179–184. 10.1097/00003086-200402000-00029 15021151

[jsap70072-bib-0090] Spencer, D.D. & Daye, R.M. (2018) A prospective, randomized, double‐blinded, placebo‐controlled clinical study on postoperative antibiotherapy in 150 arthroscopy‐assisted tibial plateau leveling osteotomies in dogs. Veterinary Surgery, 47, E79–E87. 30267441 10.1111/vsu.12958

[jsap70072-bib-0091] Stavroulaki, E.M. , Suchodolski, J.S. & Xenoulis, P.G. (2023) Effects of antimicrobials on the gastrointestinal microbiota of dogs and cats. Veterinary Journal, 291, 105929. 36427604 10.1016/j.tvjl.2022.105929

[jsap70072-bib-0092] Stetter, J. , Boge, G.S. , Grönlund, U. & Bergström, A. (2021) Risk factors for surgical site infection associated with clean surgical procedures in dogs. Research in Veterinary Science, 136, 616–621. 33905955 10.1016/j.rvsc.2021.04.012

[jsap70072-bib-0093] Swaffield, M.J. , Molloy, S.L. & Lipscomb, V.J. (2020) Prospective comparison of perioperative wound and pain score parameters in cats undergoing flank vs midline ovariectomy. Journal of Feline Medicine and Surgery, 22, 168–177. 30950672 10.1177/1098612X19837038 PMC10814559

[jsap70072-bib-0094] Turk, R. , Singh, A. & Weese, J.S. (2015) Prospective surgical site infection surveillance in dogs. Veterinary Surgery, 44, 2–8. 10.1111/j.1532-950X.2014.12267.x 25196800

[jsap70072-bib-0095] Vasseur, P.B. , Paul, H.A. , Enos, L.R. & Hirsh, D.C. (1985) Infection rates in clean surgical procedures: a comparison of ampicillin prophylaxis vs a placebo. Journal of the American Veterinary Medical Association, 187, 825–827. 3902758

[jsap70072-bib-0096] Vegas Cómitre, M.D. , Cortellini, S. , Cherlet, M. , Devreese, M. , Roques, B.B. , Bousquet‐Melou, A. et al. (2021) Population pharmacokinetics of intravenous amoxicillin combined with clavulanic acid in healthy and critically ill dogs. Frontiers in Veterinary Science, 8, 1338. 10.3389/fvets.2021.770202 PMC8636140 34869739

[jsap70072-bib-0097] von Pfeil, D.J.F. , Kowaleski, M.P. , Glassman, M. & Dejardin, L.M. (2018) Results of a survey of veterinary orthopedic society members on the preferred method for treating cranial cruciate ligament rupture in dogs weighing more than 15 kilograms (33 pounds). Journal of the American Veterinary Medical Association, 253, 586–597. 30110219 10.2460/javma.253.5.586

[jsap70072-bib-0098] Wemmers, A.C. , Charalambous, M. , Harms, O. & Volk, H.A. (2022) Surgical treatment of cranial cruciate ligament disease in dogs using tibial plateau leveling osteotomy or tibial tuberosity advancement – a systematic review with a meta‐analytic approach. Frontiers in Veterinary Science, 9, 1004637. 36532339 10.3389/fvets.2022.1004637 PMC9748159

[jsap70072-bib-0099] Whittem, T.L. , Johnson, A.L. , Smith, C.W. , Schaeffer, D.J. , Coolman, B.R. , Averill, S.M. et al. (1999) Effect of perioperative prophylactic antimicrobial treatment in dogs undergoing elective orthopedic surgery. Journal of the American Veterinary Medical Association, 215, 212–216. 10416474

[jsap70072-bib-0100] Woelber, E. , Schrick, E.J. , Gessner, B.D. & Evans, H.L. (2016) Proportion of surgical site infections occurring after hospital discharge: a systematic review. Surgical Infections, 17, 510–519. 27463235 10.1089/sur.2015.241

[jsap70072-bib-0101] Wright, E. , Jessen, L.R. , Tompson, A. , Rutland, C. , Singleton, D. , Battersby, I. et al. (2024) Influencing attitudes towards antimicrobial use and resistance in companion animals‐the impact on pet owners of a short animation in a randomized controlled trial. JAC Antimicrobial Resistance, 6, dlae065. 38716404 10.1093/jacamr/dlae065 PMC11073752

[jsap70072-bib-0102] Yang, F. , Yang, F. , Wang, G. , Xi, W. , Zhang, C. & Wang, H. (2019) Pharmacokinetics of the amoxicillin‐clavulanic acid combination after intravenous and oral administration in cats. Journal of Veterinary Pharmacology and Therapeutics, 42, 511–517. 31162674 10.1111/jvp.12765

[jsap70072-bib-0103] Yap, F.W. , Calvo, I. , Smith, K.D. & Parkin, T. (2015) Perioperative risk factors for surgical site infection in tibial tuberosity advancement: 224 stifles. Veterinary and Comparative Orthopaedics and Traumatology, 28, 199–206. 25757496 10.3415/VCOT-14-09-0141

[jsap70072-bib-0104] Zeng, L. , Brignardello‐Petersen, R. , Hultcrantz, M. , Mustafa, R.A. , Murad, M.H. , Iorio, A. et al. (2022) GRADE guidance 34: update on rating imprecision using a minimally contextualized approach. Journal of Clinical Epidemiology, 150, 216–224. 35934265 10.1016/j.jclinepi.2022.07.014

