# Supplementary material for: European Network for Optimization of Veterinary Antimicrobial Therapy (ENOVAT) 2025 guidelines for surgical antimicrobial prophylaxis in dogs and cats
Source: J Small Anim Pract. 2025 Dec 23;67(5):383–405. doi: 10.1111/jsap.70072 (PMC13136061; doi:10.1111/jsap.70072)
Supplement: Supplementary file 2 — Table S1. Summary of findings for all PICOs relating to peri‐operative SAP compared to no SAP in dogs and cats [file JSAP-67-383-s002.docx]

| **Peri-op AM compared to no AM for SAP in dogs and cats** | | | | | |
| --- | --- | --- | --- | --- | --- |
| **Outcomes** | **№ of participants (studies) Follow-up** | **Certainty of the evidence (GRADE)** | **Relative effect (95% CI)** | **Anticipated absolute effects** | |
|  |  |  |  | **Risk with no AM** | **Risk difference with Peri-op AM** |
| SSI, P1 | 835 (1 RCT) | ⨁⨁◯◯ Low | **RR 0.88** (0.57 to 1.37) | 93 per 1,000 | **11 fewer per 1,000** (40 fewer to 34 more) |
| SSI, P1 | 1343 (3 non-randomised studies) | ⨁◯◯◯ Very low | **RR 0.58** (0.23 to 1.45) | 40 per 1,000 | **17 fewer per 1,000** (31 fewer to 18 more) |
| SSI, P2 | 835 (1 RCT) | ⨁◯◯◯ Very low | **RR 0.88** (0.57 to 1.37) | 93 per 1,000 | **11 fewer per 1,000** (40 fewer to 34 more) |
| SSI, P2 | 1343 (3 non-randomised studies) | ⨁◯◯◯ Very low | **RR 0.58** (0.23 to 1.45) | 40 per 1,000 | **17 fewer per 1,000** (31 fewer to 18 more) |
| SSI, P3-P5 | 38 (1 RCT) | ⨁◯◯◯ Very low | **RR 1.35** (0.25 to 7.19) | 111 per 1,000 | **39 more per 1,000** (83 fewer to 688 more) |
| SSI, P3 | 964 (2 non-randomised studies) | ⨁◯◯◯ Very low | **RR 0.66** (0.24 to 1.85) | 77 per 1,000 | **26 fewer per 1,000** (58 fewer to 65 more) |
| SSI, P4-P5 | 964 (2 non-randomised studies) | ⨁◯◯◯ Very low | **RR 0.66** (0.24 to 1.85) | 77 per 1,000 | **26 fewer per 1,000** (58 fewer to 65 more) |
| SSI, P6 | 16 (1 non-randomised study) | ⨁◯◯◯ Very low | **RR 0.56** (0.04 to 7.83) | 286 per 1,000 | **126 fewer per 1,000** (274 fewer to 1,951 more) |
| SSI, P7-P9 | 314 (3 RCTs) | ⨁◯◯◯ Very low | **RR 0.38** (0.13 to 1.13) | 54 per 1,000 | **34 fewer per 1,000** (47 fewer to 7 more) |
| SSI, P7-P8 | 1776 (2 non-randomised studies) | ⨁◯◯◯ Very low | **RR 0.62** (0.29 to 1.33) | 50 per 1,000 | **19 fewer per 1,000** (35 fewer to 16 more) |
| SSI, P9 | 1776 (2 non-randomised studies) | ⨁◯◯◯ Very low | **RR 0.62** (0.29 to 1.33) | 50 per 1,000 | **19 fewer per 1,000** (35 fewer to 16 more) |
| ***The risk in the intervention group** (and its 95% confidence interval) is based on the assumed risk in the comparison group and the **relative effect** of the intervention (and its 95% CI).  **CI:** confidence interval; **RR:** risk ratio; SSI: surgical site infection; RCT; randomized control trial; AM: antimicrobial | | | | | |
| **GRADE Working Group grades of evidence** **High certainty:** confidence that the true effect lies close to that of the estimate of the effect. **Moderate certainty:** moderately confidence in the effect estimate: the true effect is likely to be close to the estimate of the effect, but there is a possibility that it is substantially different. **Low certainty:** the confidence in the effect estimate is limited: the true effect may be substantially different from the estimate of the effect. **Very low certainty:** very little confidence in the effect estimate: the true effect is likely to be substantially different from the estimate of effect. | | | | | |

Supplementary materials Table 1: Summary of findings for all PICOs relating to peri-operative SAP compared to no SAP in dogs and cats.
