# Supplementary material for: European Network for Optimization of Veterinary Antimicrobial Therapy (ENOVAT) 2025 guidelines for surgical antimicrobial prophylaxis in dogs and cats
Source: J Small Anim Pract. 2025 Dec 23;67(5):383–405. doi: 10.1111/jsap.70072 (PMC13136061; doi:10.1111/jsap.70072)
Supplement: Supplementary file 3 — Table S2. Summary of findings for all PICOs relating to post‐operative SAP compared to no SAP in dogs and cats [file JSAP-67-383-s003.docx]

| **Post-op AM compared to no AM for SAP in dogs and cats?** | | | | | |
| --- | --- | --- | --- | --- | --- |
| **Outcomes** | **№ of participants (studies) Follow-up** | **Certainty of the evidence (GRADE)** | **Relative effect (95% CI)** | **Anticipated absolute effects** | |
|  |  |  |  | **Risk with no AM** | **Risk difference with Post-op AM** |
| SSI, P1 | 492 (1 RCT) | ⨁◯◯◯ Very low | **RR 0.07** (0.00 to 1.18) | 28 per 1,000 | **26 fewer per 1,000** (28 fewer to 5 more) |
| SSI, P1 | 97 (1 non-randomised study) | ⨁◯◯◯ Very low | **RR 3.94** (0.24 to 64.48) | 0 per 1,000 | **0 fewer per 1,000** (0 fewer to 0 fewer) |
| SSI, P2 | 492 (1 RCT) | ⨁◯◯◯ Very low | **RR 0.07** (0.00 to 1.18) | 28 per 1,000 | **26 fewer per 1,000** (28 fewer to 5 more) |
| SSI, P2 | 97 (1 non-randomised study) | ⨁◯◯◯ Very low | **RR 3.94** (0.24 to 64.48) | 0 per 1,000 | **0 fewer per 1,000** (0 fewer to 0 fewer) |
| SSI, P3-P6 | 492 (1 RCT) | ⨁◯◯◯ Very low | **RR 0.07** (0.00 to 1.18) | 28 per 1,000 | **26 fewer per 1,000** (28 fewer to 5 more) |
| SSI, P3 | 72 (1 non-randomised study) | ⨁◯◯◯ Very low | **RR 1.18** (0.18 to 7.94) | 51 per 1,000 | **9 more per 1,000** (42 fewer to 356 more) |
| SSI, P4 | 15 (1 non-randomised study) | ⨁◯◯◯ Very low | **RR 0.50** (0.07 to 3.85) | 333 per 1,000 | **167 fewer per 1,000** (310 fewer to 950 more) |
| SSI, P5-P6 | 184 (1 non-randomised study) | ⨁◯◯◯ Very low | **RR 1.99** (0.59 to 6.73) | 52 per 1,000 | **51 more per 1,000** (21 fewer to 296 more) |
| SSI, P7 | 149 (1 RCT) | ⨁⨁◯◯ Low | **RR 1.48** (0.25 to 8.60) | 27 per 1,000 | **13 more per 1,000** (20 fewer to 205 more) |
| SSI, P8 | 149 (1 RCT) | ⨁⨁⨁◯ Moderate | **RR 1.48** (0.25 to 8.60) | 27 per 1,000 | **13 more per 1,000** (20 fewer to 205 more) |
| SSI, P9 | 467 (3 RCTs) | ⨁◯◯◯ Very low | **RR 0.39** (0.14 to 1.08) | 167 per 1,000 | **102 fewer per 1,000** (143 fewer to 13 more) |
| SSI, P9 | 253 (2 non-randomised studies) | ⨁◯◯◯ Very low | **RR 0.36** (0.16 to 0.82) | 128 per 1,000 | **82 fewer per 1,000** (107 fewer to 23 fewer) |
| ***The risk in the intervention group** (and its 95% confidence interval) is based on the assumed risk in the comparison group and the **relative effect** of the intervention (and its 95% CI).  **CI:** confidence interval; **RR:** risk ratio; SSI: surgical site infection; RCT; randomized control trial; AM: antimicrobial | | | | | |
| **GRADE Working Group grades of evidence** **High certainty:** confidence that the true effect lies close to that of the estimate of the effect. **Moderate certainty:** moderately confidence in the effect estimate: the true effect is likely to be close to the estimate of the effect, but there is a possibility that it is substantially different. **Low certainty:** the confidence in the effect estimate is limited: the true effect may be substantially different from the estimate of the effect. **Very low certainty:** very little confidence in the effect estimate: the true effect is likely to be substantially different from the estimate of effect. | | | | | |

Supplementary materials Table 2: Summary of findings for all PICOs relating to post-operative SAP compared to no SAP in dogs and cats.
