# Supplementary material for: European Network for Optimization of Veterinary Antimicrobial Therapy (ENOVAT) 2025 guidelines for surgical antimicrobial prophylaxis in dogs and cats
Source: J Small Anim Pract. 2025 Dec 23;67(5):383–405. doi: 10.1111/jsap.70072 (PMC13136061; doi:10.1111/jsap.70072)
Supplement: Supplementary file 1 — Data S1. Response to comments during consultation phase [file JSAP-67-383-s001.docx]

**Supplementary Materials:**

**Response to reviews submitted during the consultation phase**

We appreciated the reviewers’ comments and have revised the manuscript as outlined here. We have tried to defend the approach adopted and address comments where the panel felt that revisions to the manuscript could not be adopted.

We have addressed the comments from 10 reviewers, point by point below.

1. **Reviewer comment –** *Refreshing and inspiring in a broad European/EU context where tradition and routine differs a lot from the proposed guidelines!
   Just a few thoughts below:

   Inclusion of infection protection and control (Hospital Hygiene) in slightly greater detail than the current manuscript, even though those topics are said to be beyond the scope of the article's focus would really be good. For some of the recommendations flawless IPC is crucial for both reducing SSI and thus the need for antimicrobial therapy. SAP and IPC are linked!

   We often demand higher level of evidence to change behaviour than to continue "like we always have", even when the support for our routines lack, evidence-vice. Adding comments about the lack of evidence for many current SAP strategies in addition to those in the reviewed articles could perhaps be good way to account for possible critics arguing that the evidence for the recommendations are too low.*

**Author response –** Thank you for these comments.

The importance of infection prevention and control (IPC) is recognised by the guideline panel and was incorporated into the section: “Good Practice statements around the correct administration of surgical antimicrobial prophylaxis.” The panel are keen to emphasise the need for strict surgical asepsis and rigorous hospital hygiene to mitigate SSI risk. Unfortunately, manuscript size limits more detailed discussion of IPC practices. It is a very important area and a superficial overview would not do it service. Therefore, we feel that it is appropriate to raise the issue but avoid detailed discussion.

The science of inciting behaviour change is indeed highly complicated (Kelly and Barker, 2016). The panel hope that the ENOVAT guidelines will serve as a framework for national organisations to drive initiatives locally that will support rational SAP. The evidence behind each recommendation has been carefully evaluated (and graded) in this manuscript which accompanies a systematic review of the wider literature. Future work will try to maximise the impact of these recommendations through the development of infographics, translations into different languages and defining target levels for SAP for practitioners.

2. **Reviewer comment –** *I believe that in certain orthopaedic procedures a postoperative course of antibiotics is necessary (such as in total hip replacements).*

**Author response –** The authors have highlighted that all veterinary surgeons should evaluate the decision to use (or withhold) SAP carefully on a case-by-case basis. Patient factors and procedure factors may influence these decisions. These guidelines offer recommendations based on the best evidence currently available in the veterinary domain. Unfortunately, there is often a lack of procedure-specific evidence so recommendations are based on findings from other procedures deemed as similar as possible (in this case orthopaedic procedures involving the placement of an implant excluding TPLOs). The rationale for the strong recommendation not to use post-operative SAP for orthopaedic procedures involving the placement of an implant (that by definition includes total hip replacements) is defended as robustly as possible.

Of note, there is similar uncertainty in the human literature with some studies favouring extended post-operative SAP for hip or knee arthroplasty (Inabathula et al., 2018; Mohiuddin et al., 2024) while others found no supportive evidence for continuation of SAP beyond 24 hours (Kuo et al., 2019; van Kasteren et al., 2007; Villa et al., 2023; Zastrow et al., 2020). Post-operative SAP is not recommended in human guidelines (Bratzler et al., 2013) although this is indirect evidence for the considerations here. From a mechanistic standpoint, there is also no clear value of post-operative (>24h) antimicrobials as the typically assumed ‘period of risk’ is over. If bacterial contamination from that period has not been eliminated with antimicrobials, there is little reason to think that an extended duration would result in any different effect. Ultimately, though, guidelines are guidance, not standards, and local factors may influence case-level decisions. Yet, based on the available data, the committee could not make a recommendation for post-operative antimicrobials when balancing data regarding benefits and harms (no matter how limited the data may be).

3. **Reviewer comment –** *There are some orthopaedic surgeries which have a high infection rate (for example carpal arthrodesis or limb sparing procedures) which would benefit from a course of postoperative antibiotics.*

**Author response –** Similar to the previous response, the panel did not identify robust evidence in the veterinary literature that demonstrates a significant benefit from extending prophylactic antibiotic therapy beyond 24 hours (i.e., post-operative SAP). Carefully designed studies (ideally RCTs) that address these questions would greatly benefit the profession and provide greater clarity around these decisions. It is important to recognize that higher SSI rates do not necessarily mean a greater need for antimicrobials. Some procedures are inherently higher risk and antimicrobials are designed to reduce that to as low a level as possible. Antimicrobials will never completely eliminate the risk, and SSIs will still occur with prolonged courses. In the absence of direct or indirect evidence supporting a need for post-operative antimicrobials, we have to balance the potential benefits with the potential and known harms of adverse effects and resistance. Indeed, there is reasonable concern that prolonged post-operative antimicrobials could simply select for resistant rather than susceptible infections. The ability to prevent infections in patients that are inherently at high risk for infection is uncertain. While infection percentage (SSI rate) is often used as a benchmark for surgical outcomes, it does not tell the whole story. From a broader antimicrobial stewardship perspective, it may be preferable to accept a slightly higher SSI rate caused by bacteria that remain susceptible to treatment, rather than aiming for marginally lower infection rates at the potential cost of selecting for highly resistant organisms. In this context, a modest increase in easily treatable infections could be considered a more manageable outcome than a smaller number of infections that are significantly more difficult and resource-intensive to address due to resistance.

4. **Reviewer comment –** *The wording of the document needs to be changed for consistency of language. For example, some sections say 'we suggest not to use...' others say 'we recommend against the use...' is this a deliberate change in terminology with one meant to be 'stronger' than the other or is this an error?*

**Author response –** The language follows the structure outlined in the GRADE framework. The word ‘recommend’ (whether that be for or against any particular intervention) is used for strong recommendations. Where a conditional recommendation has been reached the statements include the word ‘suggest’. Efforts have been made to maintain consistency of terminology in accordance with the relevant strength of recommendation.

5. **Reviewer comment –** *Great job for this important topic. I would add a recommendation for orthopaedic cases where surgical revision is performed (during the same procedure or the day after). Also, if possible, I would suggest to precise if "perioperative" antibiotics administered after the surgery are really included in the recommendation (for example in clean-contaminated gastrointestinal procedures or clean orthopaedic procedures, why not recommend only antibioprophylaxis and eventually administrations during surgery but not after at all (not during the 24 next hours)).*

**Author response –** Thank you for these valuable suggestions. No revisional/corrective surgical procedures were included in these guidelines for several reasons. The need for and nature of revision will be specific to the pet and procedure. The timing of revision in relation to the original surgery may also vary considerably. Furthermore, the guideline document is already very lengthy (with 18 separate PICOs) prohibiting inclusion of some interesting procedures.

The recommendation to use peri-operative SAP should not be interpreted as a recommendation to continue antibiotics for 24 hours. In most cases, a single pre-operative dose? (30-60 minutes prior to incision) will be all that is required. However, there are some prolonged procedures that may warrant further antibiotic administration to ensure continued maintenance of effective antimicrobial concentrations at the surgical site. The panel have adopted a definition of peri-operative SAP that is used in human medicine and facilitates the bold statement not to use post-operative SAP for any procedure.

6. **Reviewer comment –** *Some typos are present but I guess they'll be amended before publication (recommendation 12 "we suggest we suggest that post-operative surgical", recommendation 15 "Level of agreement 100X%").*

**Author response –** Thank you for spotting these typos. The manuscript has been carefully reviewed again but occasional errers may remain.

7. **Reviewer comment –** *Antimicrobial prophylaxis means per definition that antimicrobials should be give prior to surgery, and discontinued after surgery. It is unclear why recommendation 12 includes postoperative use of antimicrobials 3-5 days after surgery.*

**Author response –** Antimicrobial prophylaxis may be continued post-operatively with prophylactic intent as there is no known infection but merely a desire to avoid that possibility. The panel elected to include contaminated procedures (PICOs 11 and 12) for completeness although arguably such antimicrobial use may be better defined as therapeutic since these are procedures that are deemed to have a high likelihood of infection developing (e.g., following spillage of gastrointestinal contents into the abdomen) or already have existing infection at/near the surgical site.

The proposed duration of antimicrobial administration is arguable, inherently arbitrary and case-dependent. There may be situations where the degree of contamination and/or tissue damage warrants >48 hours of treatment. Panel members routinely advocate a maximum continuation of antibiotic therapy for only 4 days after source control for septic peritonitis, questioning the suggestion of 3-5 days here. However, we have so little information currently, and this could be a high risk scenario that a few days is justifiable. The panel acknowledges that we don’t know the optimal recommendation here. In lieu of data, the recommendation errs on the side of likely excessive duration because of the potential for profound clinical consequences should infection develop.

8. **Reviewer comment –** *It is very nice to see these recommendations are made, however, it is equally important to add which antimicrobials should be given, the dosage, interval and route of administration.*

**Author response –** This information has been included towards the end of the main document in the sections: agent selection and dosing regimens. A short summary table has been prepared and added to the executive summary. It is important to note that the suggestions offered for preferred antimicrobials, doses and intervals have not undergone the same rigorous review process as the main recommendations. This has been acknowledged in the manuscript also. Nonetheless, it was felt that this information (already presented at the end of the guideline) could also be appended to the executive summary in a readily accessible format.

9. **Reviewer comment –** *This feedback is on behalf of researchers within the Asia Pacific Centre for Animal Health (APCAH) at the University of Melbourne.

   Suggestions based on discussion within the team:

   - Depending on the audience, it would be good to make specific recommendations for which antimicrobial to use for surgical prophylaxis (including dose rates).*

**Author response –** This information has been included towards the end of the main document in the sections: agent selection and dosing regimens.

**9b. Reviewer comment –** *Was this draft sent to specialists only and if so, why not general practitioners?*

**Author response –** The draft guidelines were made available for review during the consultation phase (January 26th- April 30^th^) via a variety of channels (via the ENOVAT website, ECVS mail server and at a number of national and international congresses) with both specialists and general practitioners encouraged to provide feedback.

9c. **Reviewer comment –** *Unclear on whether this draft is intended to represent format of future guidelines or just to inform the development of guidelines
--> if informing future guideline development, including dose rates will be important for increasing their use by clinicians*

**Author response –** The intention if for this document to serve as a reference guideline for SAP in cats and dogs. The recognised knowledge gaps highlight that future revisions will be required once more robust evidence is available to support or refute each recommendation. The panel hopes that national bodies will use these recommendations to develop their own local guidelines. It is anticipated that local guidelines, presented in the appropriate language and incorporating consideration of relevant national legislation, antimicrobial availability and IPC processes, may be developed using the ENOVAT guidelines as a base framework.

Dosing information has been included towards the end of the main document in the sections: agent selection and dosing regimens.

9d. **Reviewer comment –** *Potentially good to include a caveat about local resistance rates and laws regarding which drugs are registered and accessible*

**Author response –** These caveats have been included in the section on dosing regimens and represent one of the main reasons for not offering specific agents for each procedure.

9e. **Reviewer comment –** *Potentially change layout of results to increase clarity*

**Author response –** Efforts have been made to optimise the clarity of these guidelines especially via the inclusion of an executive summary that is hopefully more accessible to the time-poor practitioner.

9f. **Reviewer comment –** What is the justification for 3-5 days of antimicrobial therapy? Could this be shortened considering it is currently 48hrs in humans?

**Author response –** See also response to comment 7. Duration of post-operative antimicrobial therapy (and it should be considered therapeutic rather than prophylactic as discussed above), is uncertain and the panel could find little evidence to support an optimal duration. The panel would always recommend the shortest course that achieves the intended outcome. More research is required to establish what such a course would be.

9g. **Reviewer comment –** *If this is going to general practitioners, please provide more clarity on which surgeries fall under which categories and potentially include specific drugs (including examples of what general practitioners get lots of)*

**Author response –** Additional examples of procedures have been added to a table (included in Supplementary materials) for each recommendation to help practitioners as suggested.

9h. **Reviewer comment –** *Many of the recommendations are conditional and the table explaining this was difficult to understand*

**Author response –** The terminology used is derived from the GRADE framework. The inclusion of the table is intended to serve as an additional explanation. This has been reworded to improve clarity.

9i. **Reviewer comment –** *If possible, further clarity on the DELPHI, including which conditions were included, may be valuable to inform future suggestions*

**Author response –** The focus of the manuscript is on the recommendations. The panel recognises the need for transparency in the methodology to allow repeatability but must balance this with the burgeoning word count. During early drafting group meetings, different procedures, procedure types and procedure classifications were discussed. The preliminary list of procedures was reduced via an iterative Delphi process whereby the drafting group members selected preferred options, eventually arriving at the 9 procedure-groups included here.

9j. **Reviewer comment –** Recommendation 3
*- In relation to dermal mass removal, is this only if skin intact, what about if it is ulcerated, infected?
- How to distinguish or classify?*

**Author response –**

Our intention was to refer to non-infected, non-ulcerated dermal masses, typically classified as clean procedures. We have revised the recommendation to specify this more clearly, e.g.:

“Dermal mass removals are considered clean procedures when there is no clinical evidence of infection, ulceration, or contamination. In cases where the mass is ulcerated or overtly infected, the procedure should be classified as *dirty*, and antimicrobial use (if required) should be considered therapeutic rather than prophylactic.”

Regarding surgical excision of infected masses, we acknowledge that complete excision of the infection focus may negate the need for antimicrobial therapy, provided there is no evidence of local tissue spread or systemic involvement. However, if surrounding tissues show signs of infection (e.g., cellulitis, emphysema, exudate), antimicrobial treatment would be warranted.

9k. **Reviewer comment** Recommendation 5
*- In relation to clean-contaminated urological surgical procedures – please provide examples of what these procedures are and are not.*

**Author response –** Clean-contaminated urological surgical procedures could include nephrectomy, ureteric surgery (e.g., ureterotomy, ureterostomy), cystoscopic or open surgery of the urinary bladder (cystotomy), placement of a subcutaneous ureteral bypass (SUB) device, ectopic ureter reimplantation. Should urine culture be positive, these procedures would be re-classified as contaminated.

9l. **Reviewer comment** Recommendation 11
*- For peri-operative antimicrobial use in contaminated procedures, provide more specific examples
- How would pyometra be classified?*

**Author response –** This comment highlights a limitation of the current classification system. Peri-operative SAP is recommended to be initiated prior to first incision and thus cannot be applied to cases of gastrointestinal spillage (or spillage from other abdominal viscera e.g., gall bladder or urinary bladder) as this cannot be predicted before the start of the procedure. This limitation is recognised in the guidance and is one of the reasons for suggesting continued post-operative SAP in such cases. Other procedures described in human medicine as being contaminated include open cardiac massage and acute gun-shot injuries. Pyometra has not been included in these guidelines as this is typically considered a dirty procedure. The need for antimicrobial use in pyometra is the subject of ongoing discussion (Bertero et al., 2024; Lavin and Maki, 2023; Turkki et al., 2023) and research (Ylhäinen et al., 2023).

9m. **Reviewer comment** Recommendation 13
*- Expand on what is defined as an implant.
- De Angelis suture? Does it depend on the suture material?*

**Author response –** An implant may be considered anything used to replace, support or enhance an existing biological structure. Sometimes these are degradable but most often they are permanent (metallic, ceramic, or plastic) materials. The panel have endeavoured to limit reference to specific implant types where possible.

10. **Reviewer comment –** *Concerning recommendation 15: I think we should look more carefully in this. There are differences of implants and surgery types. A tuberositas tibia avulsion and a TPLO are surgeries in the same anatomical area using implants, but we all agree that for a TPLO we need SAP, but not for a tension band in the tuberositas. There are more papers documenting surgeries with implants without SAP (Tibial Tuberosity Transposition Stabilization Using a Cortical Screw Placed Adjacent to the Tuberosity in Dogs with Patellar Luxation, VCOT Open 2019;2:e43–e49; Evaluation of Surgical Technique and Clinical Results of a Procedure-Specific Fixation Method for Tibial Tuberosity Transposition in Dogs: 37 Cases, Vet Comp Orthop Traumatol 2023;36:266–272; Tibial tuberosity advancement in small-breed dogs using TTA Rapid implants: complications and outcome, Journal of Small Animal Practice (2017) 58, 314–323; Complications and Outcome of Cranial Cruciate Ligament Disease in Small Dogs Treated with Tiny TTA Rapid, VCOT Open 2023;00:1–7).*
    *More studies are necessary, of course, but we can see that a general recommendation to use SAP in implant surgeries is not correct. Time of the surgery has to be taken in consideration, type of approach and soft tissue damage.*

**Author response –** The panel agrees that there is a mixture of findings for procedures in this group. Undoubtedly, it is a limitation to include procedures with such a range of different complexity and anticipated duration in a single recommendation. Surgeons that refer to these guidelines are reminded of the need to review each decision on a case-by-case basis and may interpret the recommendations differently depending on the situation. This is the basis of a conditional recommendation. Unfortunately, the evidence was not considered sufficiently robust to offer a strong recommendation in either direction. The references mentioned above were reviewed as part of the wider scoping review but did not feature in the systematic review and as such were incorporated in the wider body of evidence rather than in the Forest plots. The panel agrees with the points raised: procedure duration, surgical approach and anticipated soft tissue damage are all important factors that can influence the decision to use or withhold peri-operative SAP. The recommendation is not a general recommendation to use SAP in all implant surgeries but a conditional recommendation that should take into account all the factors mentioned among others.

**References:**

Allerton, F., 2020. Driving laboratory standardisation of bacterial culture and antimicrobial susceptibility testing in veterinary clinical microbiology in Europe and beyond. Journal of clinical microbiology. https://doi.org/10.1128/jcm.02572-20

Bertero, A., Corrò, M., Spagnolo, E., Nervo, T., Rota, A., 2024. Antimicrobials administration, treatment outcome and bacterial susceptibility in canine pyometra cases subjected to ovariohysterectomy. Vet J 306, 106157. https://doi.org/10.1016/j.tvjl.2024.106157

Bratzler, D.W., Dellinger, E.P., Olsen, K.M., Perl, T.M., Auwaerter, P.G., Bolon, M.K., Fish, D.N., Napolitano, L.M., Sawyer, R.G., Slain, D., Steinberg, J.P., Weinstein, R.A., American Society of Health-System Pharmacists (ASHP), Infectious Diseases Society of America (IDSA), Surgical Infection Society (SIS), Society for Healthcare Epidemiology of America (SHEA), 2013. Clinical practice guidelines for antimicrobial prophylaxis in surgery. Surg Infect (Larchmt) 14, 73–156. https://doi.org/10.1089/sur.2013.9999

Inabathula, A., Dilley, J.E., Ziemba-Davis, M., Warth, L.C., Azzam, K.A., Ireland, P.H., Meneghini, R.M., 2018. Extended Oral Antibiotic Prophylaxis in High-Risk Patients Substantially Reduces Primary Total Hip and Knee Arthroplasty 90-Day Infection Rate. J Bone Joint Surg Am 100, 2103–2109. https://doi.org/10.2106/JBJS.17.01485

Kelly, M.P., Barker, M., 2016. Why is changing health-related behaviour so difficult? Public Health 136, 109–116. https://doi.org/10.1016/j.puhe.2016.03.030

Kuo, F.-C., Aalirezaie, A., Goswami, K., Shohat, N., Blevins, K., Parvizi, J., 2019. Extended Antibiotic Prophylaxis Confers No Benefit Following Aseptic Revision Total Hip Arthroplasty: A Matched Case-Controlled Study. J Arthroplasty 34, 2724–2729. https://doi.org/10.1016/j.arth.2019.06.012

Lavin, L.E., Maki, L.C., 2023. Antimicrobial use in the surgical treatment of canine pyometra: A questionnaire survey of Arizona-licensed veterinarians. Vet Med Sci 9, 1124–1133. https://doi.org/10.1002/vms3.1130

Mohiuddin, A., Rice, J., Ziemba-Davis, M., Meneghini, R.M., 2024. Infection Rates After Aseptic Revision Total Hip Arthroplasty With Extended Oral Antibiotic Prophylaxis. J Am Acad Orthop Surg 32, 472–480. https://doi.org/10.5435/JAAOS-D-23-00750

Turkki, O.M., Sunesson, K.W., den Hertog, E., Varjonen, K., 2023. Postoperative complications and antibiotic use in dogs with pyometra: a retrospective review of 140 cases (2019). Acta Vet Scand 65, 11. https://doi.org/10.1186/s13028-023-00670-5

van Kasteren, M.E.E., Manniën, J., Ott, A., Kullberg, B.-J., de Boer, A.S., Gyssens, I.C., 2007. Antibiotic prophylaxis and the risk of surgical site infections following total hip arthroplasty: timely administration is the most important factor. Clin Infect Dis 44, 921–927. https://doi.org/10.1086/512192

Villa, J.M., Pannu, T.S., Braaksma, W., Higuera, C.A., Riesgo, A.M., 2023. Extended Oral Antibiotic Prophylaxis After Aseptic Total Hip or Knee Arthroplasty Revisions: A Preliminary Report. J Arthroplasty 38, 141–145. https://doi.org/10.1016/j.arth.2022.08.003

Ylhäinen, A., Mölsä, S., Grönthal, T., Junnila, J., Rantala, M., Laitinen-Vapaavuori, O., Thomson, K., 2023. A double-blinded randomized placebo-controlled non-inferiority trial protocol for postoperative infections associated with canine pyometra. BMC Veterinary Research 19, 77. https://doi.org/10.1186/s12917-023-03629-w

Zastrow, R.K., Huang, H.-H., Galatz, L.M., Saunders-Hao, P., Poeran, J., Moucha, C.S., 2020. Characteristics of Antibiotic Prophylaxis and Risk of Surgical Site Infections in Primary Total Hip and Knee Arthroplasty. J Arthroplasty 35, 2581–2589. https://doi.org/10.1016/j.arth.2020.04.025
